# The implementation of disaster preparedness training integration model based on Public Health Nursing (ILATGANA-PHN) to increase community capacity in natural disaster-prone areas

**DOI:** 10.1186/s12912-024-01755-w

**Published:** 2024-02-07

**Authors:** Haris Sofyana, Kusman Ibrahim, Irvan Afriandi, Erna Herawati

**Affiliations:** 1 Doctoral Program, Faculty of Medicine, Padjadjaran University, Bandung, 45363 Indonesia; 2 Department of Medical and Surgical Nursing, Faculty of Nursing, Padjadjaran University, Bandung, 45363 Indonesia; 3 Departement of Public Health, Faculty of Medicine, Padjadjaran University, Bandung, 45363 Indonesia; 4 Department of Anthropology, Faculty of Social and Political Sciences, Padjadjaran University, Bandung, 45363 Indonesia

**Keywords:** Disaster, ILATGANA, PHN, Preparedness, Training

## Abstract

Indonesia is at high risk of disasters. Therefore, nursing is expected to play a role in disaster risk reductions in communities. This study aimed to implement the Disaster Preparedness Training Integration Model based on Public Health Nursing (ILATGANA-PHN) to increase the Capacity of community in natural disaster-prone areas by assessing the preparedness level of families and communities in disaster-prone areas. The research method was developed in two stages, including the model preparation stage and the model implementation stage. This research was in the 2nd stage, namely the model impelementation stage. The research design, at the model implementation stage, used the one-sample pre-post test without control group design. The respondents were assessed before and after the ILATGANA-PHN training intervention. The sample size was calculated using the sample size calculation formula for the experimental research design without controls. The samples of the study were 78 people. The result of the research described the ILATGANA-PHN training concepts, including the instrument, curriculum, process, module, and maintenance patterns for the training process. The intervention had a significant effect on increasing the independent preparedness of the people in Kendeng Community, Sugih Mukti Village (ƿ 0.000 ≤ 0.005) in terms of four preparedness parameters, namely knowledge and attitudes about disasters (KA), disaster preparedness plans (PE), disaster warnings (WS), and resource mobilization community (RMC). Nurses have the opportunity to take responsibilities for empowering the community capacity in the disaster area through the implementation of ILATGANA-PHN training. The integrated training model for disaster preparedness based on Public Health Nursing (ILATGANA-PHN) is effective in increasing the community capacity, especially in disaster managements, in disaster-prone areas.

**Supplementary Information:**

The online version contains supplementary material available at 10.1186/s12912-024-01755-w.

## Introduction

Indonesia has a lot of areas with a high frequency of natural disasters. An overview of the high incidence of disasters in Indonesia can be identified from the statistics on disaster events from 2020 to 2022. Throughout 2020, there were 3.544 disaster events (an average of 10 disaster events/day). Whereas, in 2021, it increased to 5.402 disaster events (an average of 15 disaster events/day). However, in 2022, it slightly decreased to 4650 (an average of 13 disaster events/day) [1]. The 2021 Disaster Risk Index (IRB) statistical data recorded around 5.4 thousand natural disasters occurred in Indonesia, increased from 2.5 thousand events in 2018. More than 21 thousand residential buildings were severely damaged by natural disasters occurred in Indonesia in 2021 [2]. In addition, until March 2022, non-natural disasters due to the Covid-19 pandemic still became a problem [3].

The 2021 Indonesian Disaster Risk Index calculation results showed that 15 provinces were in the high disaster risk category, 19 were in the medium disaster risk category, and no province was in the low disaster risk category [1]. West Java Province has a vulnerable geographical and topographical area to potential disasters. Spatially, almost all areas in West Java are in the high-level of disaster-prone area category. There are 4,465 villages/sub-districts in the high-level of disaster risk category out of 5,957 villages/sub-districts. This means that 75% of the total villages in West Java are in the high-level of disaster-prone area category [4].

Kendeng Community, located in Sugih Mukti Village, Bandung Regency, West Java, is a disaster-prone area because of its geographical, social, and geological factors. The main disasters that might occur are earthquakes, landslides, and gas poisoning. Kendeng Community is located at an altitude of 1200–2300 m above the sea level, right at the peak of Mount Patuha which is directly adjacent to PT Geo Dipa Energi. Geographically, it is located next to PT Geo Dipa Energi (Persero). Regionally, the Kendeng Community area has a high level of disaster risk, such as earthquakes, landslides, gas poisoning, and tornadoes [5]. The result of research shows that the major disaster threats in the Sugih Mukti Village area are earthquakes, landslides, and hurricanes [6]. This finding is also supported by a research explaining that Sugih Mukti Village is vulnerable to disasters due to its geographical structure [5].

Based on these issues, the policy orientation for the implementation of disaster management in Indonesia in the 2020–2024 period is to increase the disaster management resilience to achieve a lasting prosperity for a sustainable development [7]. In line with this policy orientation, the key factors to increase the disaster management resilience include the community preparedness and the Capacity level to protect themselves from the disaster threat. Disaster education aims to increase the understanding of risks through disaster preparedness trainings. It is an awareness process to increase their ability to save themselves. This awareness process is useful for everyone to understand the risks, to cope with the threats, and to develop the community resilience. In addition, social cohesion, mutual cooperation, and mutual trust are the glue values of social capital that have been tested and continue to be fostered in both the ability of individuals and society as a whole to prepare for, respond to, and recover from adversity caused by disasters [8].

The results of previous research showed the need for a model. There are three main problems faced by European Union member countries related to disaster risk reduction science and policy, namely knowledge transfer, disaster expertise, and risk awareness [9]. The results of research in Lambung Village, Banda Aceh, highlighted the importance of the community preparedness to reduce the risk of large number of victims [10]. Apart from that, understanding the mechanisms of local communities in recognizing disasters is the first step that needs to be taken to create a community-based disaster mitigation system. Disaster mitigation efforts can also be carried out by increasing the capacity of local communities. One of the causes of a non-optimal disaster mitigation is the low understanding of the community knowledge, suggesting the importance of the community preparedness to reduce the risk of large numbers of victims [11]. The disaster risk assessment showed a weak relation to preparedness interests. The results of the Focus Group Discussion (FGD) illustrated that the most prominent cultural characteristics involved family values ​​and the strong social cohesion; personal values ​​could be transformed into cultural values and those values ​​could support or oppose the motivation to prepare for disasters [12].

As community health professionals, nurses have a strategic role to play in supporting the community resilience. Community empowerment regarding DRR requires an inter-professional approach that is community-centered and focuses on modeling the role of trainers, nurses, and caring officers [13]. Nurse is an important part of the health profession. Understanding nurse experiences in disaster areas can help identify problems in providing nursing services in the disaster area. It can be overcome with a better planning and preparation [14]. Nurses have a major role in preparing and managing care media during disaster events. On a global scale, nurses are active participants in caring for victims in various kinds of disasters [15]. The primary nursing care practice at the community level has touched on specific areas of nursing science. For this reason, it is necessary to develop a community training program that originates from personal strengths to reach and facilitate other community groups. There is still a lack of standard guidelines to increase the community capacity in dealing with disasters and emergencies resulting from disasters in the health care sector, so that efforts to make people aware that they must be able to help themselves and others are still ineffective. Therefore, a model is needed to develop the community capacity in disaster managements.

A community empowerment model is needed in a disaster risk reduction oriented to the needs of independent community health services in the health sector. Disaster events will almost certainly cause health crises such as casualties, injuries, refugees, health problems, the availability of clean water, sanitation, environmental health, nutrition, mental health, and paralyzed health services. It requires a serious and comprehensive handling from various sectors [13]. The role of the health sector is important, especially the health professionals who provide direct services in the community. One of these health professions is nurse. The ILATGANA-PHN training model (*Integrasi Latihan Siaga Bencana*)*,* known as disaster preparedness training integration-Public Health Nursing, has been tested through the following stages: expert consultation, curriculum workshop, training implementation, and training evaluation, resulting in a training curriculum and training module draft [16].

This study aimed to implement a disaster preparedness training integration model based on Public Health Nursing (ILATGANA-PHN) to increase the community preparedness in areas prone to natural disasters. More specifically, this research aimed to 1) implement an integrated program of disaster preparedness training based on Public Health Nursing (PHN) to increase the Capacity of community in disaster-prone areas, 2) identify the preparedness level of communities in disaster-prone areas in implementing the disaster preparedness, and 3) identify the effect of various preparedness variables in supporting the integrated preparedness in disaster management preparedness programs in disaster-prone areas.

## Material and methods

The research was carried out for 1 year, from January-December 2022. The implementation stage carried out included the preparation and development of research completeness (January-March), preparation of training models (April-June 2022), implementation of the ILATGANA training (July–October 2022), and report of research results (November–December 2022). The data collection using the ILATGANA training model was carried out for two weeks, from 18 October to 1 November 2022. Meanwhile, the ILATGANA-PHN training was held on 21–23 October 2022.

### Research design

**Fig. 1:**
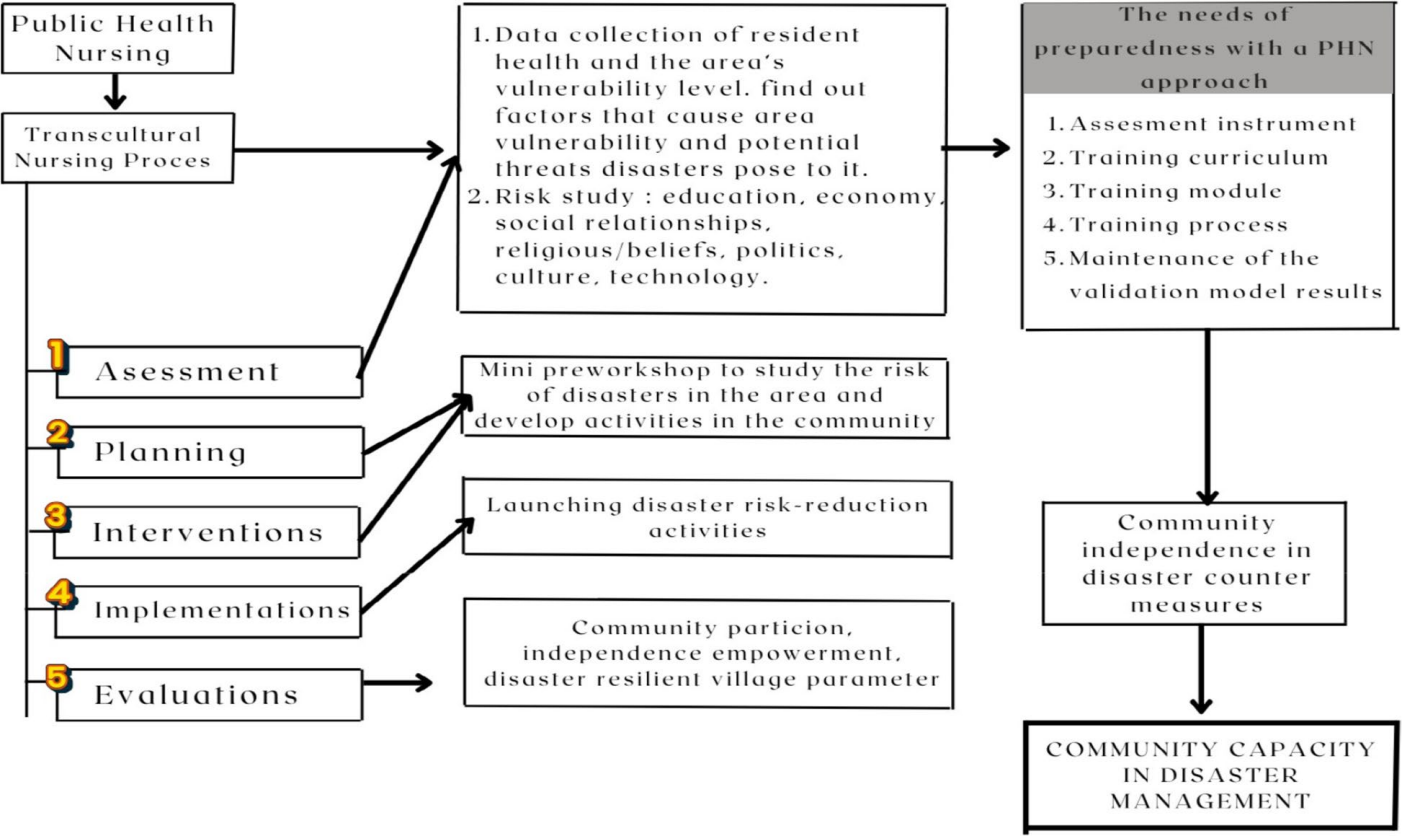
The ILATGANA PHN model construction developed by Sofyana et al. [17]

The research method was developed in two stages, namely the model development stage and the model implementation stage. The model development stage was carried out based on the model construction developed by Sofyana et al. [5, 17] in previous research (Fig. 1).

The model development was carried out in three stages, namely expert consultations (expert panel), the ILATAGANA-PHN model construction, and the ILATGANA-PHN training concept preparation. The expert panel stage involved three disaster experts from the Regional Disaster Management Agency (known as BPBD) of West Java Province, the Central Leadership Council of the Indonesian Emergency and Disaster Care Association (known as DPP HIPGABI), and disaster nursing academics/practitioners from Airlangga University, Surabaya.

The implementation of the ILATGANA-PHN model was carried out by applying the ILATGANA-PHN model to communities in the high disaster risk areas. The training was used as a medium to strengthen the knowledge, skills, and attitudes of individuals, families, and communities as disaster risk reduction cadres.

The research, at the model implementation stage, employed a one-sample pre-test without control design. The respondents were assessed before and after the ILATGANA-PHN training intervention. It is expected that the level of personal, family, and community understanding and Capacity after the intervention would be different or affected by the training.

### Population and sample

The population of this study was the Kendeng Community, Sugih Mukti Village, Pasir Jambu District, which was considered to have a high disaster potential. The population of Kendeng Community consisted of 478 people distributed in 78 heads of families (known as KK). The sample size was calculated using the sample size calculation formula for the experiment design without controls [18]. Based on a similar study conducted by [19] concerning the effect of disaster management training on the average value and deviation score of the behavior before and after the intervention, the required samples were 21 respondents. Based on the calculations, the samples were selected through a non probability purposively selected sampling by taking one representative from each family, resulting in a sample size of 78 people.

### Research instruments

The research instruments used was a respondent biodata questionnaire and a family and community Capacity questionnaire. The respondent biodata questionnaire contained a list of open questions that should be filled in by the respondents, including name (initials), place and date of birth/age, sex, experience of attending training, and experience of being a volunteer. The individual/family capacity questionnaire was the instrument for measuring the results of the ILATGANA-PHN training regarding the preparedness level.

The preparedness level of individuals/families/communities was measured by a modified instrument validated in research conducted by LIPI and UNESCO/ISDRR containing 25 question items and 104 statements. This instrument consisted of the parameters used, including knowledge and attitudes about disasters (Knowledge and Attitude/KA) consisting of 12 question items and 50 statements, family disaster preparedness plans (Emergency Planning/EP) consisting of three question items and 21 statements, disaster warning (Warning System/WS) consisting of 19 question items and 19 statements, and resource mobilization community (RMC) consisting of 5 question items and 14 statements [20]. The statements were in a Likert scale, yes and no. Furthermore, the data were converted to a scale of levels, namely not independent (1), less independent (2), independent (3), and very independent (4). The scoring results were cumulative ranged from 0–100. The level of family and community capacity was measured based on the cumulative score of these four parameters.

### Ethical approval declarations

*Approval*: All experimental protocols in this study have been approved and passed the ethical clearance test and received approval from the Institute for Ethics Studies of the Health Polytechnic of The Health Ministry, Bandung (No. 01/KEPK/EC/II/2022).

*Accordance*: This research was conducted in accordance with the ethical standards as set out in the 1964 Declaration of Helsinki and its later amendments or comparable ethical standards.

*Informed consent (for experiments involving humans or human tissue samples)*: include a statement confirming that informed consent was obtained from all participants and/or their legal guardian/s.

### Data collection and analysis

Data were collected using the respondent biodata instrument and the individual/personal Capacity questionnaire. The biodata instrument was completed by the respondent. The level of individual/personal/household Capacity was also completed by the respondents according to the level of understanding achieved, covering four aspects including knowledge and attitudes towards disasters (KA), emergency planning (EP), warning system (WS) and resource mobilization community (RMC). Each item was scored from 1 to 4.

Univariate analysis was carried out on the numerical data measuring the central tendency for age, knowledge, and attitude variables. Meanwhile, category data for sex used percentage distributions. Bivariate analysis was used to test differences in average scores before and after the training for each variable (the level of individual, family, and community Capacity) for community groups. The statistical analysis used were the independent two-average difference test and the dependent paired t-test to analyze changes in the Capacity level of individuals, families, and communities to the community disaster risk reduction assistance program (PRBOM). There were three post-intervention measurements. Each measurement was taken two weeks after the intervention.

### Model construction

#### Development of the ILATGANA-PHN model

The model development was carried out in three stages, namely the expert consultation (expert panel) stage, the ILATAGANA-PHN model construction stage, and the ILATGANA-PHN training concept development stage.

##### Expert panel for the construction of the ILATGANA-PHN model

Expert consultations or expert panels were carried out based on the results of Sofyana et al. research on model construction [5]. An expert consultation was held on July 2022. The expert consultation material was the ILATGANA-PHN model construction framework. The prepared model construction was then discussed with experts from the Indonesian Emergency and Disaster Nursing Association (HIPGABI), the Regional Disaster Management Agency (BPBD) of West Java Province, and academics in the field of disaster and emergency nursing (Airlangga University Surabaya). The results of the expert panel are presented in the following table:

#### The development of the ILATGANA-PHN training concept

##### Training instrument

The Instrument used for measuring the effect of training was the instrument developed by LIPI/UNESCO/ISDRR containing four aspects of measurement, namely knowledge and attitudes about disasters (KA), family disaster emergency planning (EP), warnings system (WS), and resource mobilization community (RMC). Meanwhile, family and community Capacitys were collected from the cumulative score of the four parameters.

##### Training curriculum

The ILATGANA-PHN training curriculum was developed as the application of the preparedness training model integrated with the PHN stages. Curriculum development was carried out by involving various influential parties in disaster management efforts, including the Regional Disaster Management Agency (BPBD), professional nurse organizations (PPNI/HIPGABI), community leaders in disaster areas, cross-cultural and religious community leaders, government officials, and non-governmental organizations. The ILATGANA-PHN training model curriculum used the Simulation Based Learning (SBL) method to comprehensively internalize the training material received. The expected competence gained in this training was the mastery of ILATGANA-PHN training materials and practices in the cognitive, affective, and psychomotor domains of the trainees so that they would be able to implement disaster management independently. ILATGANA-PHN Training Materials consisted of 28 sessions for 3 (three) days with a duration of 45 min for each session.

##### Training modules

The prepared and developed training modules consisted of theoretical learning contents, discussions, practices, and simulations. The modules were developed based on the curriculum demands and the study of the Self-Introspection Survey (SMD) results. The followings are the materials developed in the ILATGANA-PHN training module:

1. The concept of disaster and community-based disaster risk reductions
2. Community assistance and empowerment in disaster risk reductions in health
3. The role of the community in disaster risk reductions, including basic life support and basic self-rescue techniques, basic trauma and open wound managements, basic management of fluids and respiratory emergencies, basic measurement of vital signs, basic evacuation techniques
4. Contingency plans, rehearsals, and disaster management simulations by the community in health
5. All modules were adjusted to the ability and capacity of the community so that the depth and learning outcomes of each material were set for the general public.

##### Training process

**Fig. 2:**
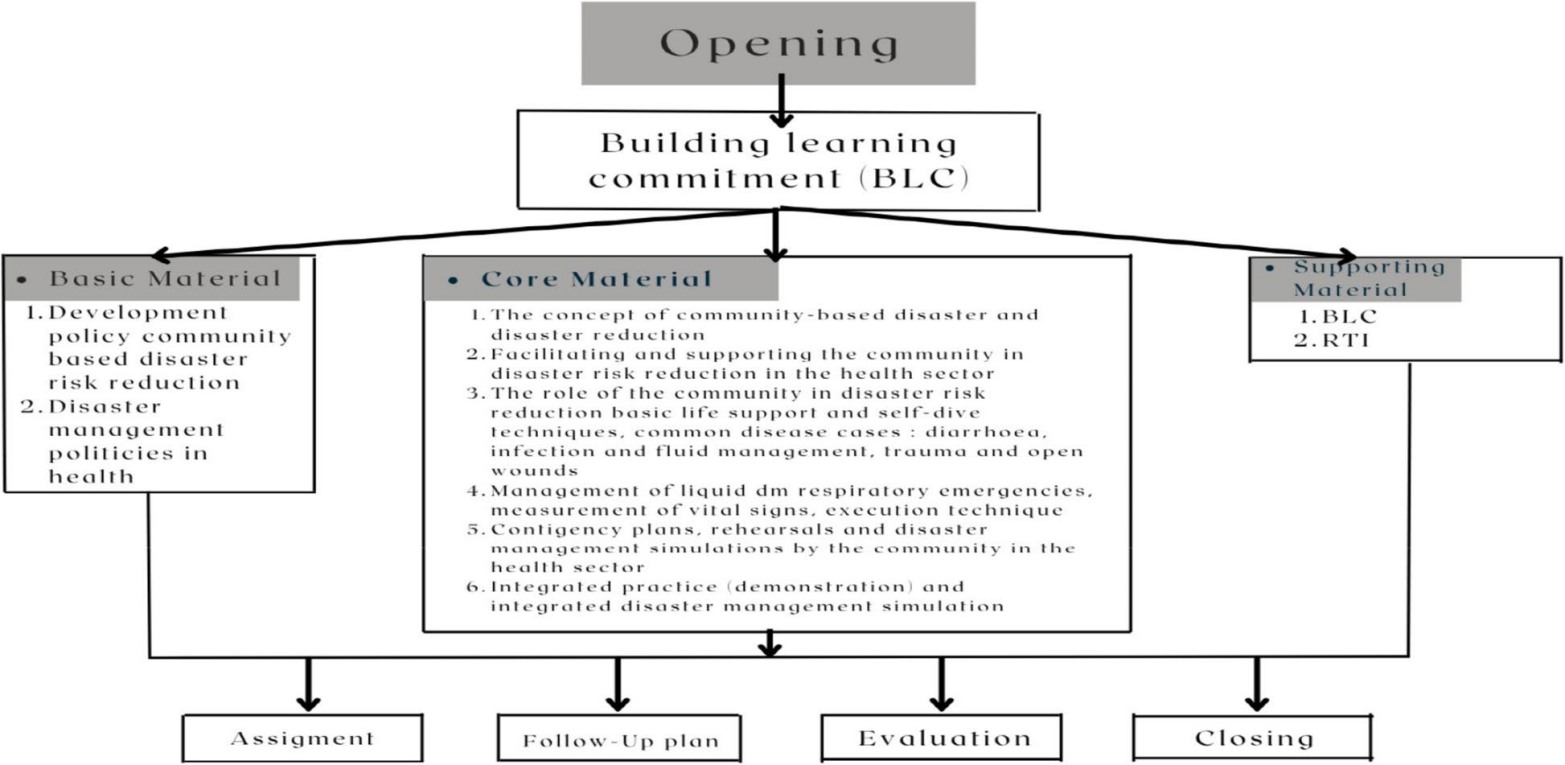
ILATGANA-PHN Training Process Scheme

The training process was carried out on 21st – 23rd October 2022. The independent training was carried out in groups at the training location. The training process was carried out to complete 28 sessions in 3 (three) days with a duration of 45 min for each session. The method used was the Simulation Based Learning (SBL), consisting of material delivery, discussions, assignments, tabletop exercises, presentations, rehearsals, and simulations. The training process scheme is shown in Fig. 2.

##### The Maintenance evaluation of the independent preparedness process

To maintain the training results, this ILATGANA-PHN training included a training maintenance process by providing a refresher training, including an assistance for one month after the training. The first mentoring was carried out two weeks after the training, namely on November 4, 2022. Discussions and evaluations of the training results were carried out in the refreshing process. The second mentoring was carried out on November 18, 2022. As in the first stage of refreshing, discussions and evaluations of the training results were also administered in the second mentoring. Then, the follow-up of the training results was carried out with the first-level health care providers, known as Puskesmas, and local government officials.

## Results

### The implementation of the ILATGANA-PHN model

The implementation of the ILATGANA-PHN model was carried out by applying the ILATGANA-PHN model intervention to communities in the high-level of disaster risk areas. The intervention was carried out on 80 selected research respondents in Kendeng Community, Sugih Mukti Village, Bandung Regency.

#### Respondent characteristics.

Based on Table 2, the sex of the respondents was evenly distributed, with a higher proportion of female participants, namely 44 people (55%).

Most of them had never been exposed to disaster management trainings, namely 50 people (64.1%). Based on the age group classification, the largest proportion was in the 46–57-year age group, namely 24 people (30.8%). Most of the participants, 60 people (76,9%), were volunteers. Based on the participant occupations, most of them, 46 people (59%), were plantation workers.

### The effect of the ILATGANA-PHN Training on the community capacity

Capacity was measured by examining the effect of ILATGANA-PHN training on the four Capacity parameters recommended by LIPI and UNESCO/ISDR, namely knowledge and attitude (KA), emergency planning (EP), disaster warning (WS), and resource mobilization community (RMC).

#### Knowledge and Attitude (KA)

**Fig. 3:**
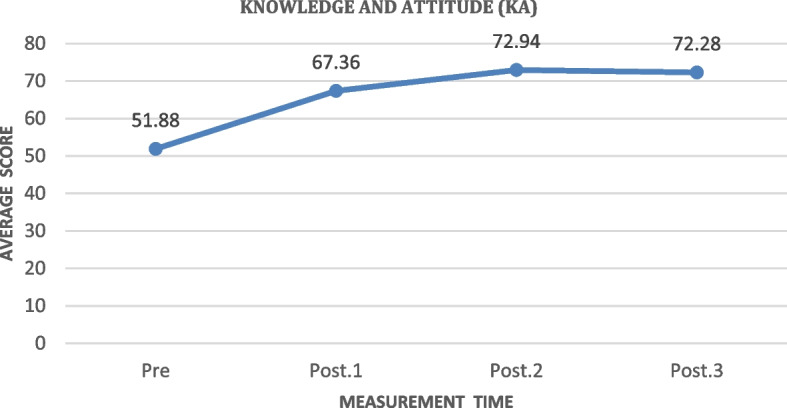
The increase of knowledge and attitude (KA) average score before and after the training in measurements 1, 2, and 3 (*N* = 78)

Based on Fig. 3, the knowledge and attitude (KA) score of the people in Kendeng Community, Sugih Mukti Village, increased from 53.81, before the training, to 70.76 one month after the training (post-test 3). The highest increase was 9.75, gained from the pre-test to post-test 1 measurement. There was also an increase of 0.07 from the post-test 3 to the post-test 4 measurement.

Table 3 shows that the ILATGANA-PHN training has a significant effect on increasing the knowledge and attitudes of the people of Kendeng Community, Sugih Mukti Village (ƿ 0.000 ≤ 0.005).

Family Emergency Planning (EP)

**Fig. 4:**
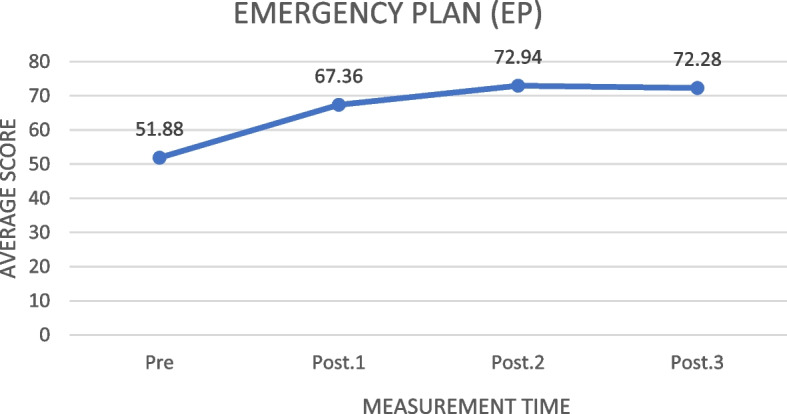
The increase of the family emergency planning (EP) average score before and after the training in measurements 1, 2, and 3 (*N* = 78)

Based on Fig. 4, the score of the 

#### Family Emergency Planning (EP)

community Capacity in emergency situation (emergency planning/EP) increased from 51.77, before the training, to 72.03 one month after the training. The highest increase appeared in the first and second measurements (16.81). From the third to the fourth measurement, there was a decrease of 1.39.

Table 4 shows that the ILATGANA-PHN training has a significant effect on increasing the emergency planning (EP) of the people at Kendeng Community, Sugih Mukti Village (ƿ 0.000 ≤ 0.005).

#### The understanding of Warning System (WS)

**Fig. 5:**
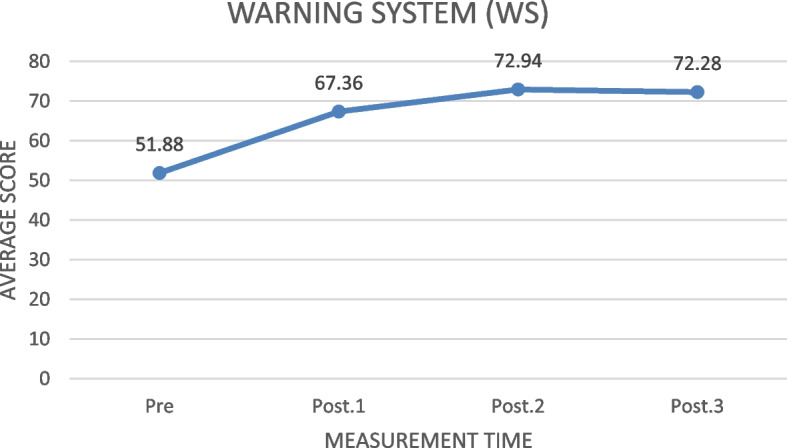
The increase of the understanding of warning system (WS) average score before and after the training in measurements 1, 2, and 3 (*N* = 78)

Based on Fig. 5, the community warning system aspect score (WS) increased from 50.41, before the training, to 71.23 one month after the training. The highest score increase was seen in the first and second measurements (15.58). From the third to the fourth measurement, there was a decrease of 1.06.

Table 5 shows that the ILATGANA-PHN training has a significant effect on improving the disaster warning system aspect of the Kendeng Community, Sugih Mukti Village (ƿ 0.000 ≤ 0.005).

#### Resource Mobilization Community (RMC) during disasters

**Fig. 6:**
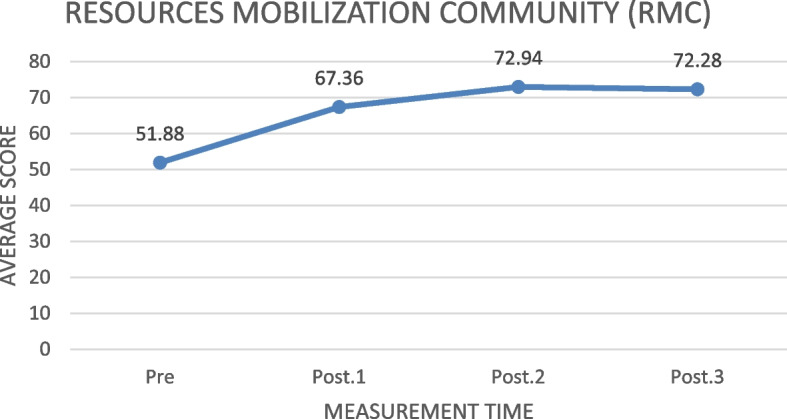
The increase of resource mobilization community (RMC) average score before and after the training in measurements 1, 2, and 3 (*N* = 78)

Based on Fig. 6, the score of the resource mobilization community (RMC) aspect increased from 51.53, before the training, to 75.09 one month after the training. The highest score increase was seen from the first to the second measurements (19.78). From the third to the fourth measurement, there was a decrease of 0.26.

Table 6 shows that the ILATGANA-PHN training has a significant effect on increasing the family and community resource mobilization (RMC) in Kendeng Community, Sugih Mukti Village (ƿ 0.000 ≤ 0.005).

#### The effect of the ILATGANA-PHN training on the level of the community capacity

The level of Capacity was measured from the cumulative score of the four community capacity parameters. The effect of the four preparedness parameters is presented as follows:

Based on Table 7, the knowledge and attitude (KA) has a significant effect on increasing the community capacity (p 0.000 ≤ 0.005) with a strength of effect (R^a^) of 0.512 at alpha 5% based on the results of the F-test. Knowledge and attitude (KA) can affect changes in the community capacity (R-square) by 0.262 (26.2%). While the remaining 73.8% is affected by other variables. It means that knowledge and attitude (KA) can simultaneously affect the community capacity by 26.2%.

The emergency planning (EP) has a significant effect on increasing the community capacity (p 0.000 ≤ 0.005) with a strength of effect (R^a^) of 0.762 at alpha 5% based on the results of the F test. The emergency planning (EP) can affect changes in the community capacity (R-square) by 0.581(58.1%). While the remaining 41.9% is affected by other variables. It means that the emergency planning (EP) can simultaneously affect the community capacity by 58.1%.

The warning system (WS) has a significant effect on increasing the community capacity (p 0.000 ≤ 0.005) with a strength of effect (R^a^) of 0.552 at alpha 5% based on the results of the F test. The warning system (WS) can affect changes in the community capacity (R-square) by 0.315 (31.5%). Meanwhile, the remaining 68.5% is affected by other variables. It means that warning system (WS) can simultaneously affect the community capacity by 31.5%.

The resource mobilization community (RMC) has a significant effect on increasing the community capacity (p 0.000 ≤ 0.005) with a strength of effect (R^a^) of 0.506 at alpha 5% based on the results of the F test. The resource mobilization community (RMC) variable can affect changes in the community capacity (R-square) by 0.256 (25.6%). Meanwhile, the remaining 74.4% is affected by other variables. It means that the resource mobilization community (RMC) can simultaneously affect the community capacity by 25.6%.

**Fig. 7:**
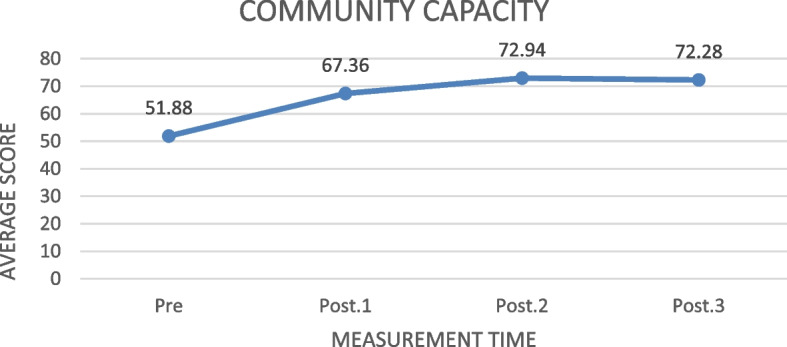
The increase of the community capacity average score level before and after the training in measurements 1, 2, and 3 (*N* = 78)

Based on Fig. 7, the highest increase of the community capacity score was from the first to the second measurements (15.48 points). In the third and fourth measurements, there was a decrease of around 0.66 points.

Table 8 shows that the ILATGANA-PHN training has a significant effect on increasing the community capacity in Kendeng Community, Sugih Mukti Village (ƿ 0.000 ≤ 0.005).

## Discussion

### The ILATGANA-PHN training model

The disaster preparedness training integration model based on public health nursing model (ILATGANA-PHN) uses community empowerment as the key element in the development of disaster management models. This training is health (nursing) training based on the social behavior of the community. It has implications for participatory social transformations. Community Health Nursing (CHN), better known as Public Health Nursing (PHN), is a field of health nursing combining nursing and public health supported by an active community participation. PHN prioritizes promotive and preventive services on a continuous basis balanced with curative and rehabilitative services and integrated services as a whole. PHN service scopes address individuals, families, groups, and communities through the nursing process to improve optimal human life functions so that they are independent in their health efforts.

Community nursing or PHN programs, to empower community groups by increasing the community Capacity, has been widely used. The research findings recommended the importance of training for empowering rural communities living in disaster-prone areas to normalize the physical and psychological problems of natural disaster victims. Community preparedness is an important key to reduce health problems resulting from natural disasters [19]. This is in line with research which showing that the empowerment process through a contingency planning approach could increase the adolescent preparedness and Capacity against the threat of death due to disasters. Therefore, empowering adolescents through contingency planning could be a recommendation to all disaster activists to increase the preparedness against the threat of death [21].

The ILATGANA-PHN training was developed as the application of the preparedness training model integrated with the implementation of PHN stages to achieve the community Capacity. The trial and development phases were carried out in the second year of the research through the implementation/replication process of several nursery areas, followed by socializations and public testing. Preparatory efforts were made with various widely involved and influential sectors and stakeholders in disaster management efforts, namely the Provincial/District/City Regional Disaster Management Authority (BPBD), nursing professional organizations (PPNI/HIPGABI), intercultural and religious community leaders, government officials, and non-governmental organizations.

The ILATGANA-PHN training model uses the Simulation Based Learning (SBL) method to comprehensively internalize the training materials received. The SBL method stimulates trainees to be more active during the training process, resulting in a stronger absorption and memory of training materials and topics. Although not exactly the same, the ILATGANA-PHN training model is in line with the model developed by Ren et al. (2017). In their research, stated that mental health training programs should identify the needs of disaster volunteers and victims. The model explains the need to use a people-centered community interprofessional education approach focusing on the role-modeling of the trainer, caring for relief workers, paying attention to the needs of the trainee, and building systematic interprofessional education strategies. The proposed model identifies areas for the comprehensive training of relief workers and aims to address the importance of people-centered mental health service provisions, ensures an intentional and strategic training of relief workers using interprofessional concepts and strategies, and uses culturally attuned and community-informed strategies in mental health training practices [13].

### The effect of the ILATGANA-PHN training on the community preparedness

#### Knowledge and Attitudes about disasters (KA)

The results showed that ILATGANA-PHN training affected the knowledge and attitudes of families and communities about a disaster. The knowledge possessed by a person or community will indirectly affect their attitudes and behavior, especially in anticipating a disaster event [22]. This is in line with the stating that one of the efforts to increase knowledge in shaping a positive attitude towards disaster events can be done through a training process. Furthermore, it emphasizes that the knowledge about a certain object will become an attitude if this knowledge is accompanied by a preparedness to act in accordance with the object. So, the attitude is a follow-up of a person knowledge about an object [23].

The effect of the ILATGANA-PHN training in increasing the knowledge and attitudes of families and communities about disasters can be explained by the fact that training can increase a person knowledge because knowledge is the result of perceiving an object [24]. These findings are in line with another research that education and training could improve preparedness in dealing with fire disasters in hospitals for nurses, so it could support the increase of knowledge and fire practices for nurses and health workers in hospitals [25].

The importance of knowledge to build Capacity and preparedness is also conveyed by another research. The community ability to organize and integrate information into existing knowledge remains poor. These problems must be solved by increasing the public knowledge of disasters, increasing disaster information media, and conducting a frequent counseling about disasters [26]. The results of other studies indicated that there was a relationship between the level of knowledge and preparedness attitudes. There was a positive relationship between the level of knowledge and attitude and the landslide disaster preparedness (*p* = 0.000, α = 0.05), *r* = 0.610. There was a unidirectional relationship between the level of knowledge and attitude and the landslide disaster preparedness [27]. The result of other research showed that the community knowledge about floods, earthquakes, and landslides in Wonogiri sub-district was in the low-medium category [28]. The preparedness attitude will ultimately promote the Capacity of action and the ability to make the right decisions in the community.

In the context of preparedness as a trigger of Capacity, the effective disaster preparedness, response, and recovery require a well-planned and integrated efforts by experienced professionals who can apply specialized knowledge and skills in critical situations. Professionals are trained for this, but others may not have the critical knowledge and experience needed to effectively perform actions under the stressful disaster conditions [29]. Preparedness can be assessed by five indicators adopted from LIPI/UNESCO ISDRRs, namely knowledge and attitudes (KA), emergency planning (EP), warning systems (WS), resource mobilization capacity (RMC), and social capital [20]. Knowledge can usually influence attitudes and concern for preparedness in anticipating disasters [30]. Meanwhile, the research conducted by Waluya & Kautsar [31] found that there was a relationship between disaster knowledge and community preparedness in facing landslides in RT001/RW002 Cibadak Village, Cibadak Sub-district, the working area of Puskesmas Sekarwangi, Sukabumi Regency.

The ILATGANA-PHN training is a reinforcement of the recommendations of the research conducted by [32]. A positive correlation was found between disaster knowledge and disaster awareness behavior in flood areas as indicated by the correlation coefficient (0.643). There was a strong positive correlation between the leadership of local leaders in the flood management and disaster awareness behaviors as evidenced by the high correlation coefficient (0.950). The strong contribution of the regional head in the flood management (90.2%) to disaster awareness behaviors was demonstrated in the investigation [33]. The results of another study concluded that there was a relationship between the community knowledge and disaster preparedness attitudes in Lambung Village, Banda Aceh. It is expected that the government will evaluate and monitor the disaster preparedness system in the community related to the lack of community knowledge [10].

#### Emergency Planning (EP)

The results showed that the ILATGANA-PHN training could improve the family and community disaster preparedness emergency plans. Planning is the key to Capacity in disaster preparedness. The planning implementation will reduce disaster risks. This result supports the policy direction of the implementation of disaster management for the 2020–2024 period, namely increasing disaster resilience to resilient welfare for a sustainable development [7]. The disaster preparedness plan component, or emergency planning (EP), strongly supports the community-based disaster risk reduction. The community-based disaster risk reduction (CBDRR) is an activity of community awareness and preparedness in identifying disaster threats and developing local action plans to reduce disaster risks independently.

In this context, disasters can occur anywhere, so a plan to avoid emergencies is an international concern. Planning for disasters is essential to consider whether a long-term community care can respond effectively to disasters. It has been found that planning for a long-term care in individuals and communities has reduced disaster risks through a collaborative community planning [34]. One of the positive comparative results that communities should independently prepare for disasters is shown in a research report. It explains that the public health programs to reduce the radiation exposure to the community after the *Fukushima Accident* that occurred on 11 March 2011 are valuable lessons for community Capacity in disaster preparedness [35].

Another research report explains that the emergency planning preparedness is a disaster risk reduction (DRR) paradigm approach. It is the right answer to make regional disaster management efforts. This risk reduction paradigm implies that every individual and community in the region is introduced to the various hazards and vulnerabilities that exist in their area and also to increase the capacity of the community in dealing with every hazard [36]. Thus, community capacity efforts in dealing with disasters can be realized.

#### The understanding of Warning System (WS)

Based on the results of the research, the ILATGANA-PHN training increased the understanding of family and community warning systems. Disaster early warning or often called warning system (WS) is a major component of disaster risk reductions. Improving the availability of multi-hazard warning systems and disaster risk information is one of the seven global targets set by The Sendai Framework for Disaster Risk Reduction 2015–2030 [37]. The results of this study are in line with one of the action plans in Indonesia disaster management strategic plan, namely developing a preparedness exercise plan (siren activation and self-evacuation) involving the population in residential neighborhoods, offices, schools, public areas, and others [7]. The ILATGANA-PHN training model is needed for the community and nurses as one of the barometers of health services. The competencies for the first-level health care providers (known as Puskesmas) include 3 categories: (1) Disaster/Emergency Preparedness System, (2) Warning System and Response, (3) Patient Care and Mass Casualty Management [38].

The United Nation Disaster Reduction recommends that the warning system is able to give clear job descriptions as a part of occupational health and safety standards in disaster-prone countries [39]. The ILATGANA-PHN training provides directions so that the community is able to independently analyze natural signs of potential disasters in an area. This is in accordance with the direction of BNPB as the Government Agency responsible for disaster issues. A warning system is a series of activities to provide an immediate warning to the public about the possibility of a disaster occurring somewhere by an authorized institution. Early warning is a part of the implementation of disaster management besides disaster preparedness and mitigation efforts (Article 44 letter b) [40]. Early warning is carried out through several stages, namely (1) observation of disaster signs, (2) analysis of the results of observations of disaster signs, (3) decision-making by the authorities, (4) dissemination of information about disaster warnings, and (5) taking action by the community. These stages are then named as the disaster early warning system [40].

#### Resource Mobilization Capacity (RMC)

The results showed that the ILATGANA-PHN training improved the ability to mobilize family resources during disasters. Resource mobilization is one of the important aspects in the disaster management. Resources are related to the Capacity of preparedness in supporting the implementation of policies or plans that have been made. Resources are related to several aspects, including human resources, budgets, facilities, information, and authority [36]. The ILATGANA-PHN training can be implemented by nurses to empower the community Capacity. In fact, in disaster emergencies, public health needs are carried out by strengthening the PHN system as health services during disasters. For this reason, the competencies required by health workers at the first level are (1) Disaster/Emergency Preparedness, Early Warning and Response System, (2) Patient Care and Mass Casualty Management, and (3) Resource (human and material) Management and Eviction [38]. In line with the results of this study, the ability to mobilize resources is closely related to the Capacity in the forest and land fire disaster preparedness. This mobilization capability includes technical guidance and material provisions, fundings and logistics, social networks, monitoring, and evaluation [41].

Based on the results of this study, nurses have the opportunity to take responsibility for empowering community resilience in the disaster field by implementing the ILATGANA-PHN training. Disaster-Related Community Resilience provides nurses with a foundation to participate in resilience-building activities that can save lives and enable communities to recover more quickly after disasters [42]. The ILATGANA-PHN training can be manifested as an implementation of the nurse existence in the community empowerment, especially in the field of disaster, because nurses can use one or more instruments to simultaneously assess aspects of resilience in the community and provide validity and reliability of data for these instruments. In addition, nurses can also take part in various disaster nursing research in disaster-related community resilience efforts. This new theoretical framework explains and predicts how social resources influence resilience at both the community and individual levels [43].

The ILATGANA-PHN training builds various community mobilization efforts in disaster management. The process of implementing the ILATGANA-PHN model emphasizing the steps of the nursing process is relevant to the explanation of Gulzar et al. (2012) that the work model of nurses in earthquake areas is carried out to empowering the community in teams with a nursing process approach in the community. Interventions are determined through a collaborative PHN process guided by a planning cycle framework. This framework includes four phases, namely assessment, planning, implementation, and evaluation. The framework provides specific directions for working with health care providers on community health promotion in a more systematic manner [44]. It is further explained that community nurses in disaster community empowerment can increase the resilience of disaster-affected communities. CHNs should focus on maximizing, preserving, and accommodating culture to maintain familiar life patterns when community circumstances are disrupted by powerful natural forces [45].

#### The effect of the ILATGANA-PHN Training on the community capacity

The results showed a strong effect of the ILATGANA-PHN training on the capacity of Kendeng Community, Sugih Mukti Village. The results of this study indicate that the disaster management training model based on the local empowerment is effective for the community Capacity in the landslide natural disaster management. The disaster management training model has a significant effect on increasing the understanding, awareness, and attitude of the community in landslide-prone areas in the pre-disaster, intra-disaster, and post-disaster phases [46].

The results of this study solve one of the problems in disaster management. One of the major issues in recent years has been the community resilience, or the ongoing ability of a community to survive and recover from disaster (e.g. economic stress, influenza pandemic, natural or man-made disaster). As resources are limited during emergencies, the community should be more aware that they should be independent after an emergency and before the help arrives, so they need to build a resilience before an emergency occurs**.** Resilience is also considered critical to a community ability to mitigate the lengthy recovery period after an emergency, which may require considerable time and resources at the federal, state, and local levels [47]. It implies that the ILATGANA-PHN training supports the preparation of resilient community capabilities in disaster managements.

The ILATGANA-PHN training model that provides an independent acceleration of community capacity in disaster managements, as the integration of key public health concepts, such as sustainability, community mobilization, community empowerment, and active community participation, is essential in intervention projects such as training. However, the type of community participation, for example in decision-making, planning, and internalizing the importance of the initiative, should be reflected by public health leaders and key stakeholders before designing the program. As a result of the interventions undertaken, few lessons were learned while working in the earthquake-affected areas. There should be a clear role for PHNs in this program. The role of nurses, in this case, will ensure an effective rehabilitation and promotion of public health in earthquake-affected areas, particularly from a nursing perspective. People from grassroots communities should be involved in the planning stage for a proper allocation of resources in disaster-affected areas. Therefore, the community participation and sustainability should be ensured from the beginning of the training process [44]. However, there is a need for a legal framework that supports and builds a culture of volunteerism among nurses to identify and prepare healthcare volunteers in the preparedness phase and assign them appropriately in the response phase**.** In addition, necessary steps to improve the retention and motivation of volunteer nurses should be prioritized. Plans should also be implemented for the volunteer dismissal and follow-up the volunteer physical and mental health after their missions [48]. PHN nurses can develop their functions and roles as health volunteers in the entire disaster management cycle.

Based on the discussion, the ILATGANA-PHN training model can be used as an option for developing the community empowerment carried out by nurses in their role in the society. It is in accordance with research results explaining that the role of nurses when disaster occurs is to manage and empower the community and facilitate the family function by involving the community in planning and implementing disaster preparedness activities in the community, as well as encouraging families to actively participate in PRB [48]. Apart from that, the government institution such as BNPB can use the ILATNAGA-PHN model as a reference for implementing the community empowerment in organizing disaster nursing service programs integrated with programs at community health centers. As we known, the Ministry of Health, through their decree in Minister of Health of the Republic of Indonesia number Hk.01.07/Menkes/16/5/2023 concerning accreditation standards for Community Health Centers, has established emergency and disaster management programs as one of the assessment parameters for the management of health facilities at Community Health Centers.

## Conclusions

The disaster preparedness training integration model based on Public Health Nursing (ILATGANA-PHN) is an integrated training developed based on the training instrument, training curriculum, training module, training process, and evaluation, which had been developed as the result of the public health nursing process. The ILATGANA-PHN has 28 sessions that involve professionals with SBL method. The ILATGANA-PHN is effective in increasing the community Capacity in disaster management in disaster-prone areas. The ILATGANA-PHN training integration implies that the training model is implemented as a whole by involving related institutions, a variety of methods, and a comprehensive empowerment process. The ILATGANA-PHN training can improve various parameters of community Capacity in disaster management, such as knowledge and attitude (KA), emergency planning (EP), warning system (WS), and resource mobilization community (RMC).

The ILATGANA-PHN training model can increase the Capacity of communities in disaster-prone areas in implementing various disaster risk reduction efforts. In this case, the role of community nurses can be expanded as disaster volunteers in the health sector who have a professional competence. The empowerment program of community Capacity in disaster managements can be synergized with various programs from policymakers, both government and private parties to strengthen this Capacity. Thus, the ILATGANA PHN training, as one of the ways of empowering nurses in disaster managements, will be more effective in increasing the community Capacity if it is facilitated by various support systems.

The ILATGANA-PHN training model will become a new reference in the field of community and disaster nursing in developing the foundational framework of nursing science. As a result, it can be considered as an alternative application of the nursing role that integrates the nursing science, disaster management, and socio-cultural aspects, specifically anthropological considerations. The ILATGANA-PHN training model is quite effective and flexible to implement. In disaster nursing, health during disasters and culture are becoming trending topics and frequent issues as the intensity of disaster happening in various regions. The ILATGANA-PHN training model is relevant to government programs in community-based disaster risk reductions.

### Supplementary materials

The following supporting information can be downloaded at: https://drive.google.com/drive/folders/1-AUJmiJvGP8qRvvTNo8JU8qNomE0L8Qb?usp=sharing Video ILATGANA_PHN Training Process, Data Availability and Graphical abstract. Figure 1: ILATGANA PHN model construction developed by Sofyana et al., [17]; Fig. 2: ILATGANA-PHN Training Process Scheme; Fig. 3: the increasing of the average score of knowledge and attitude (KA) before training and after training in measurements 1, 2, and 3; the people of Kendeng Community, Sugih Mukti Village, Bandung Regency (*N* = 78); Fig. 4: the average score increase of Family Emergency Plan (KEP) before training and after training in measurements 1, 2, and 3 Kendeng Community, Sugih Mukti Village, Bandung Regency (*N* = 78); Fig. 5: the increase of average score of Understanding on Warning System (WS) before training and after training in measurements 1, 2, and 3 Kendeng Community, Sugih Mukti Village, Bandung Regency (*N* = 78); Fig. 6: the increase of Resource Mobilization Community (RMC) average score before training and after training in measurements 1, 2, and 3 Kendeng Community, Sugih Mukti Village, Bandung Regency (*N* = 78); Fig. 7 the increase in community preparedness average score level before training and after training in measurements 1, 2, and 3 Kendeng Community, Sugih Mukti Village, Bandung Regency (*N* = 78); Table 1: Model construction expert panel resume results; Table 2. Distribution of Respondent Characteristics, Kendeng Community, Sugih Mukti Village, Bandung Regency (*N* = 78)); Table 3: The Effect of ILATGANA-PHN Training on Knowledge and Attitudes (KA) of the people of Kendeng Community, Sugih Mukti Village, Bandung Regency (*N* = 78); Table 4: Effects of ILATGANA-PHN training on Emergency Planning (EP) Kendeng Community, Sugih Mukti Village, Bandung Regency (*N* = 78); Table 5: The Effect of ILATGANA-PHN training on Warning System (WS) of Kendeng Community, Sugih Mukti Village, Bandung Regency (*N* = 78); Table 6: The effect of ILATGANA-PHN training on the Resource Mobilization Community (RMC) of Kendeng Community, Sugih Mukti Village Bandung Regency (*N* = 78); Table 7: The effect of knowledge and attitudes (KA), Emergency Planning (EP), Warning System (WS), and Resource Mobilization Community (RMC) on Kendeng Community Capacity, Sugih Mukti Village, Bandung Regency (*N* = 78); Table 8. The effect of ILATGANA-PHN training on community Capacity of Kendeng Community, Sugih Mukti Village, Bandung Regency (*N* = 78); and Video 1: ILATAGANA-PHN Training Process.

**Table 1:** Model construction expert panel resume results

EXPERT 1	EXPERT 2	EXPERT 3
Interesting and substantial research topics in disaster nursing The novelty can be identified from the substance of disaster training for people, in the field of disaster nursing, with the integration of assessments based on transcultural nursing variables The meaning of integrated training must include a minimum of a Penta helix component in a community empowerment program in the field of disaster The community resilience concept must meet clear indicators and parameters The operational implementation of ILATGANA-PHN must include themes, objectives, duties and responsibilities, scenarios, post rehearsals, tactics, and simulations The program sustainability (maintenance) should be considered	The training themes are synergistic with programs being built and developed by BPBD and the government through BNPB Please synergize with the Disaster Preparedness Village Program or Destana (BNPB, 2012) A time line at the sustainability program stage is needed Output indicators or research results can support 20 indicators of Disaster Resilient Village (Destana)	This research is good as a part of the development of nursing science It is recommended that future studies can be conducted in a wider scope The substance of the model is complete and the descriptions of the research framework and model framework are in accordance with the research objectives It is necessary to clarify the role of nurses in the integration model offered ILATGANA-PHN training must reach the simulation stage It is necessary to integrate the self-preparedness concept to save themselves from the start of the training process by referring to the major disaster potential in the research area

**Table 2:** The distribution of respondent characteristics, Kendeng Community, Sugih Mukti Village, Bandung Regency (*n*-78)

VARIABLE	F	%
Gender		
• Man	34	43,6
• Woman	44	56,4
Training experience		
• Once	28	35,9
• Never	50	64,1
Education		
• Doesn’t go to school	8	10,3
• Basic Education (Primary)	40	51,3
• Secondary Education (SMP/SMA)	30	38,4
• Higher Education (PT)	0	0
Age		
• 15 – 25 years	5	6,4
• 26 – 35 years	18	23,1
• 36 – 45 years	14	17,9
• 46 – 55 years	24	30,8
• ≥ 56 years	17	21,8
Role		
• Youth organization	7	9
• Public figure	4	5,1
• Health Cadre	5	6,4
• Government officials	2	2,6
• Volunteer	60	76,9
Work		
• Plantation Workers	46	59
• PNS/Employee	8	10,3
• Student/Students	3	3,8
• Doesn't work	21	26,9
Total	78	100

**Table 3:** The effect of the ILATGANA-PHN training on knowledge and attitudes (KA) (*N* = 78)

Variabel	Mean	SD	*P*-value
Knowledge and Attitude			
Pre test	53,81	15,52	0, 000
Post Test 1	63,56	11,73	
Post test 2	70,69	9,47	
Post Test 3	70.76	8,94

**Table 4:** The effect of the ILATGANA-PHN training on the emergency planning (EP) (*N* = 78)

Variable	Mean	Standard deviation	*P*-value
Emergency Planning (EP)			
Pre test	51,77	18,68	0, 000
Post Test 1	68,38	10,01	
Post test 2	73,42	7,82	
Post Test 3	72,03	7,56

**Table 5:** The effect of the ILATGANA-PHN training on the warning system (WS) (*N* = 78)

Variable	Mean	Standard deviation	*P*-value
Warning System (WS)			
Pre test	50,41	15,73	0, 000
Post Test 1	65,99	9,53	
Post test 2	72,29	6,48	
Post Test 3	71,23	6,82

**Table 6:** The effect of the ILATGANA-PHN training on the resource mobilization community (RMC) (*N* = 78)

Variabel	Mean	Standar deviasi	*P*-value
Resource Mobilization Community (RMC)			
Pre test	51,53	22,15	0, 000
Post Test 1	70,31	8,51	
Post test 2	75,35	6,43	
Post Test 3	75,09	7,22

**Table 7:** The effect of the knowledge and attitudes (KA), emergency planning (EP), warning system (WS), and resource mobilization community (RMC) on the community capacity (*N* = 78)

Variable	Mean	SD	n	*p*-value	R^a^	R square
Knowledge and Attitude (KA)	53.8077	15.51878	78	0,000	0,512	0,262
Community Independence	51.8365	10.59786	78			
Emergency Planning (EP)	51.7692	18.68462	78	0,000	0,762	0,581
Community Independence	51.8365	10.59786	78			
Warning System (WS)	50.4103	15.72857	78	0,000	0,552	0,315
Community Independence	51.8365	10.59786	78			
Resource Mobilization Community (RMC) Community capacity	51.5385 51.8365	22.12637 10.59786	78 78	0,000	0,506	0,256

**Table 8:** The effect of the ILATGANA-PHN training on the community Capacity of Kendeng Community, Sugih Mukti Village, Bandung Regency (*N* = 78)

Variable	Mean	Standard deviation	*P*-value
Community Independence			
Pre test	51,88	10,60	0, 000
Post Tes 1	67,38		
Post test 2	72,94		
Post test 3	72,28		

## Limitation

In this research, the knowledge and attitude variables were measured in a single variable, namely the knowledge and affective (KA) variable. Ideally, these two variables are separate variables and are measured with separate measuring instruments. However, since the instrument used is a validated instrument from research conducted by LIPI/UN/UNDRRR, it is still used as one variable.

This research did not use a comparation group or (control) as a research design. This study also did not use other models as a comparation. We call this as one sample without control design. Although, methodologically, it would be better to use a control group or other model as a comparation. As the authors, we also prepared a discussion section by comparing various research results using the community empowerment model in disaster managements.

The model is specifically used to empower the community in facing natural disaster such as earthquakes, volcanic eruptions, landslides, floods, etc., so it requires several modifications if it is going to be applied to communities with demographically different disaster potentials.

### Supplementary Information

**Additional file 1.** Basic data of ilatgana-phn research.

**Additional file 2.** Result of data analisys.

**Additional file 3. **

## Data Availability

Data is provided within the manuscript or supplementary information files.

## Abbreviations

ILATGANA: Integrasi Latihan Siaga Bencana / Disaster Preparedness Training Integration
DRR: Disaster Risk Reduction
PRB: Pengurangan Risiko Bencana / Disaster Risk Reduction
PHN: Public Health Nursing
PPNI: Persatuan Perawat Nasional Indonesia /Indonesian National Nurse Association
HIPGABI: Himpunan Perawat Gawat Darurat dan Bencana Indonesia/ Indonesian Emergency and Disaster Nurses Association
DPP HIPGABI: Dewan Pimpinan Pusat / Central Board HIPGABI
KK: Kepala Keluarga / heads of families
LIPI: Lembaga Ilmu Pengetahuan Indonesia / Indonesian Institute of Sciences
UNESCO: The United Nations Educational, Scientific and Cultural Organization
ISDRR: International Strategy For Disaster Risk Reduction

## References

[1] Adi A, Shalih O, Shabrina. Fathia, Rizqi A, Putra A. Indeks Resiko Bencana Indonesia (IRBI) Tahun 2021. Yunus R, editor.. Jakarta: Pusat Data, Informasi dan Komunikasi Kebencanaan Badan Nasional Penanggulangan Bencana 1; 2021. 8–11.

[2] Statista Research Departemen. Natural disasters in Indonesia - statistics & facts | Statista. 2021; Available from: https://www.statista.com/topics/8305/natural-disasters-in-indonesia/#dossierKeyfigures.

[3] Suryotomo P. Webinar “Peran Perawat dalam Kesiapsiagaan Bencana” oleh Persatuan Perawat Nasional Indonesia (PPNI) Tahun 2022 Kesiapsiagaan dan Peran Perawat dalam Menghadapi Bencana. In Jakarta; 2022.

[4] Supriyanto, Melilano I, Budianto A, Andreas H, Mariany A, Novianto B. Jabar Resilience Culture Province (JRCP) Cetak Biru : Jawa barat Berbudaya Tangguh Bencana. Bandung; 2021.

[5] Sofyana H, Ibrahim K, Afriandi I, Herawati E, Wahito Nugroho HS (2022). The Need for a Preparedness Training Model on Disaster Risk Reduction Based on Culturally Sensitive Public Health Nursing (PHN). Int J Environ Res Public Health.

[6] Hamzah A, Sofyana H, Cahyaningsih H, Hufad A, Hasanah VR, Handayani D (2022). Community-Based Information for Disaster Risk Identification. Disaster Emerg Med J.

[7] BNPB. Rencana Nasional Penanggulangan Bencana 2020–2024. Rencana Nasional Penanggulangan Bencana 2020–2024. 2019. 1–115 Available from: https://www.bnpb.go.id//uploads/renas/1/BUKU RENAS PB.pdf.

[8] Amri M, Robi, Yulianti, Gita, Yunus, Ridwan, et al. Risiko Bencana Indonesia (Disasters Risk of Indonesia). Badan Nasional Penanggulangan Bencana. Jakarta: BNPB. 2016;8:9–140.

[9] Albris K, Lauta KC, Raju E (2020). Disaster Knowledge Gaps: Exploring the Interface Between Science and Policy for Disaster Risk Reduction in Europe. Int J Disaster Risk Sci..

[10] Suryadi T, Zulfan Z, Kulsum K (2021). The Relationship between Knowledge and Attitudes about Community Disaster Preparedness in Lambung Village. Banda Aceh Int J Disaster Manag.

[11] Santoso MB, Buchari A, Darmawan I (2019). Mekanisme Masyarakat Lokal Dalam Mengenali Bencana Di Kabupaten Garut. Share Soc Work J.

[12] Appleby-Arnold S, Brockdorff N, Jakovljev I, Zdravković S (2018). Applying cultural values to encourage disaster preparedness: Lessons from a low-hazard country. Int J Disaster Risk Reduct.

[13] Ren ZJ, Wang HT, Zhang W (2017). Experiences in disaster-related mental health relief work: An exploratory model for the interprofessional training of psychological relief workers. J Interprof Care.

[14] Pourvakhshoori N, Norouzi K, Ahmadi F, Hosseini M, Khankeh H (2017). Nurse in limbo: A qualitative study of nursing in disasters in Iranian context. PLoS One..

[15] Veenema TG. Disaster Nursing And Emergency Preparedness. 2nd ed. New York: Springer Publishing Company; 2007. Available from: www.springerpub.com.

[16] Sofyana H. Keperawatan Gawat Darurat dan Manajemen Bencana. In: Asman A, Ruhardi A, editors. 1st ed. Tasik Malaya: Perkumpulan Rumah Cemerlang Indonesia; 2022. Available from: www.rcipress.rcipublisher.org.

[17] Sofyana H, Ibrahim K, Afriandi I, Herawati E, Wahito Nugroho HS (2022). The Need for a Preparedness Training Model on Disaster Risk Reduction Based on Culturally Sensitive Public Health Nursing (PHN). Int J Environ Res Public Health..

[18] Lameshow, Hosmer, Klar, Lwanga (1997). Besar sampel dalam penelitian kesehatan.

[19] Setiawan A, Sofyana H, Suhanda P. Health Notions , Volume 1 Issue 1 ( January-March 2017 ) ISSN 2580-4936 Empowering Village Cluster as Task Force in The Normalization of Disaster Victims ’ Physical Problems 22 | Publisher : Humanistic Network for Science and Technology Health Notions , V. 2017;1(1):22–8.

[20] Hidayati D (2015). Widayatun, Hartana P, Triyono, Kusumawati T. Panduan mengukur tingkat kesiapsiagaan masyarakat dan komunitas sekolah..

[21] Salasa S, Murni TW, Emaliyawati E (2017). Pemberdayaan pada Kelompok Remaja melalui Pendekatan Contingency Planning dalam Meningkatkan Kesiapsiagaan terhadap Ancaman Kematian Akibat Bencana. J Pendidik Keperawatan Indones.

[22] Ristiani IY (2020). Manajemen Kesiapsiagaan Dalam Menghadapi Potensi Bencana Di Kabupaten Sumedang. J Pemerintah Dan Keamanan Publik (JP dan KP).

[23] Dahniar A (2019). Memahami Pembentukan Sikap ( Attitude ). J Balai Diklat Keagamaan Bandung..

[24] Linda SE (2019). Pengaruh Pelatihan Terhadap Pengetahuan Dan Sikap Kader Kesehatan Tentang Keselamatan Pasien. J Keperawatan..

[25] Setyawan H, Nugraheni AM, Haryati S, Qadrijati I, Fajariani R, Wardani TL (2021). The correlation of fire knowledge toward disasters response and preparedness practice among hospital nurse Klaten Central Java, Indonesia. IOP Conf Ser Earth Environ Sci..

[26] Marlyono SG, Pasya GK, Nandi (2016). Peranan Literasi Informasi Bencana Terhadap Kesiapsiagaan Bencana Masyarakat Jawa Barat. Gea J Pendidik Geogr..

[27] Rini I, Kristianingrum N, Widyastikasari R (2019). Relationship Between Level Of Disaster Knowledge And Attitude Of Landslide Disaster Preparedness In Volunteers "Kelurahan Tangguh” In Malang City. J ILMU KEPERAWATAN (Journal Nurs Sci. J Journal Nurs Sci..

[28] Larasati Y, Humairoh Utami M, Dwi Pramita R, Dicky S (2017). Tingkat Pengetahuan Masyarakat Terhadap Bencana Banjir, Gempa Bumi, Dan Tanah Longsor Di Kecamatan Wonogiri. Pros Semin Nas Geogr UMS..

[29] Walsh L, Subbarao I, Gebbie K, Schor KW, Lyznicki J, Strauss-Riggs K (2012). Core competencies for disaster medicine and public health. Disaster Med Public Health Prep.

[30] Jahirin S (2021). Hubungan Pengetahuan Mitigasi Bencana dengan Kesiapsiagaan Masyarakat dalam Menghadapi Bencana Banjir. Heal J.

[31] Waluya A, Kautsar R. Hubungan Pengetahuan Tentang Mitigasi Bencana Longsor Dengan Sikap Kesiapsiagaan Masyarakat Di RT001 / RW002 Desa Cibadak Wilayah Kerja Puskesmas Sekarwangi Kabupaten Sukabumi. 2021;VII(2). p.12-19.

[32] Asadzadeh M, Aryankhesal A, Seyedin H, Babaei J (2013). The Relationship between Knowledge and Attitude of Managers with Preparedness of Healthcare Centers in Rey Health Network against Earthquake Risk-2013. Heal Emergencies Disasters Q..

[33] Sunaryo (2017). Correlation between Knowledge of Disaster, Leadership of Regional Leader and Disaster Awareness Behavior - A Correlation Study of Households in East Jakarta. Int J Sci Res..

[34] Covan EK, Fugate-Whitlock E (2010). Emergency planning and long-term care: Least paid, least powerful, most responsible. Health Care Women Int.

[35] Shimura T, Yamaguchi I, Terada H, Robert Svendsen E, Kunugita N (2015). Public health activities for mitigation of radiation exposures and risk communication challenges after the Fukushima nuclear accident. J Radiat Res.

[36] Pramono S, Yusuf M (2015). Implementasi Penanggulangan Bencana Berbasis Masyarakat ( Studi Pengembangan Penanggulangan Bencana Desa Tangguh di Desa Boboh Kecamatan Menganti ). J Ilmu Adm..

[37] Nations U (2015). Sendai Framework for Disaster Risk Reduction 2015–2030. Cmaj..

[38] Mawardi F, Lestari AS, Randita ABT, Kambey DR, Prijambada ID (2021). Strengthening Primary Health Care: Emergency and Disaster Preparedness in Community with Multidisciplinary Approach. Disaster Med Public Health Prep.

[39] Mcdonald A, Wilcox T, Aslam P, Pannawadee S, Janne P, Animesh K, et al. Disaster Risk Reduction in Indonesia Disaster Risk Reduction. 2020;40. p.14-18.

[40] Undang-undang Republik Indonesia Nomor 24 Tahun 2007. Undang-undang Republik Indonesia Nomor 24 Tahun 2007 Tentang Penanggulangan Bencana. 2007;50. p.21.

[41] Windusari F, Harjanti D, Tampubolon B. Kemampuan Mobilisasi Sumberdaya Masyarakat. Sosial Khatulistiwa: Jurnal Pendidikan IPS 2022;02(01):33–8.

[42] Heagele T (2017). Disaster-Related Community Resilience: A Concept Analysis and a Call to Action for Nurses. Public Health Nurs.

[43] Peek L, Abramson DM, Cox RS, Fothergill A. Anak-anak dan Bencana. 2017;(November). p.243-265.

[44] Gulzar SA, Faheem ZA, Somani RK (2012). Role of community health nurse in earthquake affected areas. J Pak Med Assoc.

[45] Marutani M, Harada N, Takase K, Okuda H, Anzai Y (2021). Culturally sensitive disaster nursing by Public health nurses in Japan. Public Health Nurs.

[46] Trisnamansyah. S dan Nurjanah. Model Pelatihan Penanggulangan Bencana dalam Meningkatkan Kemandirian Masyarakat : Studi di Daerah Rawan longsor Kawasan Cadas Pangeran Kabupaten Sumedang Propinsi Jawa Barat. In: No. Bandung; 2010; 15: 165–79.

[47] Chandra A, Acosta J, Stern S, Uscher-Pines L, Williams MV, Yeung D (2011). Building community resilience to disasters.

[48] Salmani I, Seyedin H, Ardalan A, Farajkhoda T (2019). Correction: Conceptual model of managing health care volunteers in disasters: A mixed method study (BMC Health Services Research (2019) 19 (241) DOI: 10.1186/s12913-019-4073-6). BMC Health Serv Res..

