# Supplementary material for: The implementation of disaster preparedness training integration model based on Public Health Nursing (ILATGANA-PHN) to increase community capacity in natural disaster-prone areas
Source: BMC Nurs. 2024 Feb 7;23:105. doi: 10.1186/s12912-024-01755-w (PMC10848510; doi:10.1186/s12912-024-01755-w)
Supplement: Supplementary file 2 — Additional file 2. Result of data analisys. [file 12912_2024_1755_MOESM2_ESM.rtf]

RESULT OF DATA ANALISYS 
Frequencies
Notes	
Output Created	31-DEC-2022 20:16:42	
Comments		
Input	Data	C:\7. Disk Terpakai INDUK\Penelitian\Penelitian 2021\Laporan Tahun Ke 2 2022\Data Hasil Pelatihan ILATAGANA.sav	
	Active Dataset	DataSet1	
	Filter	<none>	
	Weight	<none>	
	Split File	<none>	
	N of Rows in Working Data File	78	
Missing Value Handling	Definition of Missing	User-defined missing values are treated as missing.	
	Cases Used	Statistics are based on all cases with valid data.	
Syntax	FREQUENCIES VARIABLES=KAP_Pre KAP_Post1 KAP_Post2 KAP_Post3
  /STATISTICS=STDDEV MINIMUM MAXIMUM MEAN
  /ORDER=ANALYSIS.	
Resources	Processor Time	00:00:00,02	
	Elapsed Time	00:00:00,01	
Statistics	
	KAP_Pre	KAP_Post1	KAP_Post2	KAP_Post3	
N	Valid	78	78	78	78	
	Missing	0	0	0	0	
Mean	53.8077	63.5641	70.6923	70.7564	
Std. Deviation	15.51878	11.72918	9.47081	8.93583	
Minimum	4.00	40.00	47.00	47.00	
Maximum	82.00	85.00	89.00	85.00	


Frequency Table
KAP_Pre	
	Frequency	Percent	Valid Percent	Cumulative Percent	
Valid	4.00	1	1.3	1.3	1.3	
	20.00	1	1.3	1.3	2.6	
	27.00	1	1.3	1.3	3.8	
	29.00	1	1.3	1.3	5.1	
	30.00	1	1.3	1.3	6.4	
	32.00	1	1.3	1.3	7.7	
	34.00	1	1.3	1.3	9.0	
	35.00	1	1.3	1.3	10.3	
	36.00	4	5.1	5.1	15.4	
	38.00	3	3.8	3.8	19.2	
	39.00	4	5.1	5.1	24.4	
	41.00	2	2.6	2.6	26.9	
	42.00	1	1.3	1.3	28.2	
	44.00	1	1.3	1.3	29.5	
	46.00	4	5.1	5.1	34.6	
	47.00	3	3.8	3.8	38.5	
	49.00	3	3.8	3.8	42.3	
	53.00	1	1.3	1.3	43.6	
	54.00	4	5.1	5.1	48.7	
	55.00	2	2.6	2.6	51.3	
	56.00	1	1.3	1.3	52.6	
	58.00	1	1.3	1.3	53.8	
	61.00	4	5.1	5.1	59.0	
	62.00	1	1.3	1.3	60.3	
	63.00	2	2.6	2.6	62.8	
	64.00	3	3.8	3.8	66.7	
	65.00	3	3.8	3.8	70.5	
	66.00	2	2.6	2.6	73.1	
	67.00	6	7.7	7.7	80.8	
	68.00	1	1.3	1.3	82.1	
	69.00	1	1.3	1.3	83.3	
	70.00	5	6.4	6.4	89.7	
	71.00	1	1.3	1.3	91.0	
	72.00	2	2.6	2.6	93.6	
	74.00	2	2.6	2.6	96.2	
	76.00	1	1.3	1.3	97.4	
	78.00	1	1.3	1.3	98.7	
	82.00	1	1.3	1.3	100.0	
	Total	78	100.0	100.0		
KAP_Post1	
	Frequency	Percent	Valid Percent	Cumulative Percent	
Valid	40.00	1	1.3	1.3	1.3	
	41.00	1	1.3	1.3	2.6	
	45.00	2	2.6	2.6	5.1	
	46.00	1	1.3	1.3	6.4	
	47.00	1	1.3	1.3	7.7	
	48.00	1	1.3	1.3	9.0	
	49.00	2	2.6	2.6	11.5	
	50.00	4	5.1	5.1	16.7	
	51.00	3	3.8	3.8	20.5	
	52.00	4	5.1	5.1	25.6	
	53.00	2	2.6	2.6	28.2	
	54.00	1	1.3	1.3	29.5	
	55.00	4	5.1	5.1	34.6	
	56.00	1	1.3	1.3	35.9	
	58.00	2	2.6	2.6	38.5	
	59.00	1	1.3	1.3	39.7	
	61.00	4	5.1	5.1	44.9	
	63.00	2	2.6	2.6	47.4	
	64.00	2	2.6	2.6	50.0	
	66.00	1	1.3	1.3	51.3	
	67.00	5	6.4	6.4	57.7	
	68.00	1	1.3	1.3	59.0	
	69.00	1	1.3	1.3	60.3	
	70.00	3	3.8	3.8	64.1	
	71.00	4	5.1	5.1	69.2	
	72.00	3	3.8	3.8	73.1	
	73.00	1	1.3	1.3	74.4	
	74.00	4	5.1	5.1	79.5	
	75.00	3	3.8	3.8	83.3	
	76.00	3	3.8	3.8	87.2	
	77.00	1	1.3	1.3	88.5	
	78.00	1	1.3	1.3	89.7	
	80.00	2	2.6	2.6	92.3	
	81.00	2	2.6	2.6	94.9	
	82.00	3	3.8	3.8	98.7	
	85.00	1	1.3	1.3	100.0	
	Total	78	100.0	100.0		


KAP_Post2	
	Frequency	Percent	Valid Percent	Cumulative Percent	
Valid	47.00	1	1.3	1.3	1.3	
	50.00	1	1.3	1.3	2.6	
	52.00	2	2.6	2.6	5.1	
	54.00	1	1.3	1.3	6.4	
	55.00	1	1.3	1.3	7.7	
	56.00	3	3.8	3.8	11.5	
	57.00	1	1.3	1.3	12.8	
	59.00	3	3.8	3.8	16.7	
	60.00	1	1.3	1.3	17.9	
	62.00	1	1.3	1.3	19.2	
	63.00	1	1.3	1.3	20.5	
	64.00	1	1.3	1.3	21.8	
	65.00	2	2.6	2.6	24.4	
	66.00	2	2.6	2.6	26.9	
	67.00	3	3.8	3.8	30.8	
	68.00	5	6.4	6.4	37.2	
	69.00	1	1.3	1.3	38.5	
	70.00	6	7.7	7.7	46.2	
	71.00	3	3.8	3.8	50.0	
	72.00	2	2.6	2.6	52.6	
	73.00	5	6.4	6.4	59.0	
	74.00	6	7.7	7.7	66.7	
	75.00	3	3.8	3.8	70.5	
	76.00	2	2.6	2.6	73.1	
	77.00	2	2.6	2.6	75.6	
	79.00	1	1.3	1.3	76.9	
	80.00	5	6.4	6.4	83.3	
	81.00	1	1.3	1.3	84.6	
	82.00	5	6.4	6.4	91.0	
	83.00	1	1.3	1.3	92.3	
	84.00	3	3.8	3.8	96.2	
	85.00	2	2.6	2.6	98.7	
	89.00	1	1.3	1.3	100.0	
	Total	78	100.0	100.0		


KAP_Post3	
	Frequency	Percent	Valid Percent	Cumulative Percent	
Valid	47.00	1	1.3	1.3	1.3	
	50.00	1	1.3	1.3	2.6	
	53.00	1	1.3	1.3	3.8	
	55.00	1	1.3	1.3	5.1	
	56.00	4	5.1	5.1	10.3	
	57.00	2	2.6	2.6	12.8	
	59.00	1	1.3	1.3	14.1	
	60.00	3	3.8	3.8	17.9	
	61.00	1	1.3	1.3	19.2	
	62.00	1	1.3	1.3	20.5	
	64.00	2	2.6	2.6	23.1	
	65.00	1	1.3	1.3	24.4	
	66.00	1	1.3	1.3	25.6	
	67.00	4	5.1	5.1	30.8	
	68.00	2	2.6	2.6	33.3	
	69.00	3	3.8	3.8	37.2	
	70.00	3	3.8	3.8	41.0	
	71.00	4	5.1	5.1	46.2	
	72.00	5	6.4	6.4	52.6	
	73.00	2	2.6	2.6	55.1	
	74.00	6	7.7	7.7	62.8	
	75.00	2	2.6	2.6	65.4	
	76.00	5	6.4	6.4	71.8	
	77.00	2	2.6	2.6	74.4	
	78.00	4	5.1	5.1	79.5	
	79.00	3	3.8	3.8	83.3	
	80.00	2	2.6	2.6	85.9	
	81.00	6	7.7	7.7	93.6	
	83.00	1	1.3	1.3	94.9	
	84.00	2	2.6	2.6	97.4	
	85.00	2	2.6	2.6	100.0	
	Total	78	100.0	100.0		


FREQUENCIES VARIABLES=PE_Pre PE_Post1 PE_Post2 PE_Post3
  /STATISTICS=STDDEV MINIMUM MAXIMUM MEAN
  /ORDER=ANALYSIS.

Frequencies
Notes	
Output Created	31-DEC-2022 20:25:18	
Comments		
Input	Data	C:\7. Disk Terpakai INDUK\Penelitian\Penelitian 2021\Laporan Tahun Ke 2 2022\Data Hasil Pelatihan ILATAGANA.sav	
	Active Dataset	DataSet1	
	Filter	<none>	
	Weight	<none>	
	Split File	<none>	
	N of Rows in Working Data File	78	
Missing Value Handling	Definition of Missing	User-defined missing values are treated as missing.	
	Cases Used	Statistics are based on all cases with valid data.	
Syntax	FREQUENCIES VARIABLES=PE_Pre PE_Post1 PE_Post2 PE_Post3
  /STATISTICS=STDDEV MINIMUM MAXIMUM MEAN
  /ORDER=ANALYSIS.	
Resources	Processor Time	00:00:00,02	
	Elapsed Time	00:00:00,00	


Statistics	
	PE_Pre	PE_Post1	PE_Post2	PE_Post3	
N	Valid	78	78	78	78	
	Missing	0	0	0	0	
Mean	51.7692	68.5769	73.4231	72.0256	
Std. Deviation	18.68462	10.01106	7.81693	7.56096	
Minimum	5.00	48.00	57.00	48.00	
Maximum	86.00	86.00	90.00	81.00	


Frequency Table
PE_Pre	
	Frequency	Percent	Valid Percent	Cumulative Percent	
Valid	5.00	1	1.3	1.3	1.3	
	14.00	3	3.8	3.8	5.1	
	19.00	3	3.8	3.8	9.0	
	24.00	4	5.1	5.1	14.1	
	29.00	2	2.6	2.6	16.7	
	33.00	3	3.8	3.8	20.5	
	38.00	3	3.8	3.8	24.4	
	43.00	3	3.8	3.8	28.2	
	48.00	8	10.3	10.3	38.5	
	52.00	13	16.7	16.7	55.1	
	57.00	5	6.4	6.4	61.5	
	62.00	9	11.5	11.5	73.1	
	67.00	4	5.1	5.1	78.2	
	71.00	8	10.3	10.3	88.5	
	76.00	7	9.0	9.0	97.4	
	81.00	1	1.3	1.3	98.7	
	86.00	1	1.3	1.3	100.0	
	Total	78	100.0	100.0		
PE_Post1	
	Frequency	Percent	Valid Percent	Cumulative Percent	
Valid	48.00	2	2.6	2.6	2.6	
	52.00	6	7.7	7.7	10.3	
	57.00	8	10.3	10.3	20.5	
	62.00	11	14.1	14.1	34.6	
	67.00	12	15.4	15.4	50.0	
	71.00	12	15.4	15.4	65.4	
	76.00	12	15.4	15.4	80.8	
	81.00	11	14.1	14.1	94.9	
	86.00	4	5.1	5.1	100.0	
	Total	78	100.0	100.0		
PE_Post2	
	Frequency	Percent	Valid Percent	Cumulative Percent	
Valid	57.00	4	5.1	5.1	5.1	
	62.00	7	9.0	9.0	14.1	
	67.00	10	12.8	12.8	26.9	
	71.00	17	21.8	21.8	48.7	
	75.00	1	1.3	1.3	50.0	
	76.00	17	21.8	21.8	71.8	
	81.00	15	19.2	19.2	91.0	
	86.00	6	7.7	7.7	98.7	
	90.00	1	1.3	1.3	100.0	
	Total	78	100.0	100.0		
PE_Post3	
	Frequency	Percent	Valid Percent	Cumulative Percent	
Valid	48.00	1	1.3	1.3	1.3	
	52.00	1	1.3	1.3	2.6	
	57.00	2	2.6	2.6	5.1	
	62.00	9	11.5	11.5	16.7	
	67.00	9	11.5	11.5	28.2	
	71.00	22	28.2	28.2	56.4	
	73.00	1	1.3	1.3	57.7	
	76.00	13	16.7	16.7	74.4	
	81.00	20	25.6	25.6	100.0	
	Total	78	100.0	100.0		


FREQUENCIES VARIABLES=EWS_Pre EWS_Post1 EWS_Post2 EWS_post3
  /STATISTICS=STDDEV MINIMUM MAXIMUM MEAN
  /ORDER=ANALYSIS.
Frequencies
Notes	
Output Created	31-DEC-2022 20:33:42	
Comments		
Input	Data	C:\7. Disk Terpakai INDUK\Penelitian\Penelitian 2021\Laporan Tahun Ke 2 2022\Data Hasil Pelatihan ILATAGANA.sav	
	Active Dataset	DataSet1	
	Filter	<none>	
	Weight	<none>	
	Split File	<none>	
	N of Rows in Working Data File	78	
Missing Value Handling	Definition of Missing	User-defined missing values are treated as missing.	
	Cases Used	Statistics are based on all cases with valid data.	
Syntax	FREQUENCIES VARIABLES=EWS_Pre EWS_Post1 EWS_Post2 EWS_post3
  /STATISTICS=STDDEV MINIMUM MAXIMUM MEAN
  /ORDER=ANALYSIS.	
Resources	Processor Time	00:00:00,02	
	Elapsed Time	00:00:00,01	
Statistics	
	EWS_Pre	EWS_Post1	EWS_Post2	EWS_post3	
N	Valid	78	78	78	78	
	Missing	0	0	0	0	
Mean	50.4103	65.9872	72.2949	71.2308	
Std. Deviation	15.72857	9.53121	6.47695	6.82418	
Minimum	17.00	46.00	59.00	58.00	
Maximum	80.00	87.00	87.00	85.00	


Frequency Table
EWS_Pre	
	Frequency	Percent	Valid Percent	Cumulative Percent	
Valid	17.00	1	1.3	1.3	1.3	
	20.00	1	1.3	1.3	2.6	
	22.00	2	2.6	2.6	5.1	
	24.00	1	1.3	1.3	6.4	
	26.00	1	1.3	1.3	7.7	
	28.00	3	3.8	3.8	11.5	
	30.00	3	3.8	3.8	15.4	
	33.00	4	5.1	5.1	20.5	
	35.00	3	3.8	3.8	24.4	
	37.00	2	2.6	2.6	26.9	
	41.00	1	1.3	1.3	28.2	
	43.00	3	3.8	3.8	32.1	
	46.00	4	5.1	5.1	37.2	
	48.00	1	1.3	1.3	38.5	
	50.00	5	6.4	6.4	44.9	
	52.00	7	9.0	9.0	53.8	
	54.00	5	6.4	6.4	60.3	
	57.00	9	11.5	11.5	71.8	
	59.00	1	1.3	1.3	73.1	
	61.00	1	1.3	1.3	74.4	
	63.00	3	3.8	3.8	78.2	
	65.00	2	2.6	2.6	80.8	
	67.00	4	5.1	5.1	85.9	
	70.00	3	3.8	3.8	89.7	
	72.00	3	3.8	3.8	93.6	
	74.00	2	2.6	2.6	96.2	
	78.00	2	2.6	2.6	98.7	
	80.00	1	1.3	1.3	100.0	
	Total	78	100.0	100.0		
EWS_Post1	
	Frequency	Percent	Valid Percent	Cumulative Percent	
Valid	46.00	3	3.8	3.8	3.8	
	50.00	1	1.3	1.3	5.1	
	52.00	4	5.1	5.1	10.3	
	54.00	4	5.1	5.1	15.4	
	57.00	2	2.6	2.6	17.9	
	59.00	7	9.0	9.0	26.9	
	61.00	6	7.7	7.7	34.6	
	63.00	8	10.3	10.3	44.9	
	65.00	5	6.4	6.4	51.3	
	67.00	5	6.4	6.4	57.7	
	69.00	1	1.3	1.3	59.0	
	70.00	7	9.0	9.0	67.9	
	72.00	5	6.4	6.4	74.4	
	73.00	1	1.3	1.3	75.6	
	74.00	5	6.4	6.4	82.1	
	76.00	3	3.8	3.8	85.9	
	78.00	4	5.1	5.1	91.0	
	80.00	3	3.8	3.8	94.9	
	83.00	3	3.8	3.8	98.7	
	87.00	1	1.3	1.3	100.0	
	Total	78	100.0	100.0		
EWS_Post2	
	Frequency	Percent	Valid Percent	Cumulative Percent	
Valid	59.00	1	1.3	1.3	1.3	
	61.00	4	5.1	5.1	6.4	
	63.00	4	5.1	5.1	11.5	
	65.00	6	7.7	7.7	19.2	
	67.00	8	10.3	10.3	29.5	
	70.00	10	12.8	12.8	42.3	
	72.00	8	10.3	10.3	52.6	
	74.00	8	10.3	10.3	62.8	
	76.00	10	12.8	12.8	75.6	
	78.00	8	10.3	10.3	85.9	
	80.00	5	6.4	6.4	92.3	
	83.00	3	3.8	3.8	96.2	
	85.00	2	2.6	2.6	98.7	
	87.00	1	1.3	1.3	100.0	
	Total	78	100.0	100.0		
EWS_post3	
	Frequency	Percent	Valid Percent	Cumulative Percent	
Valid	58.00	1	1.3	1.3	1.3	
	59.00	3	3.8	3.8	5.1	
	61.00	4	5.1	5.1	10.3	
	63.00	6	7.7	7.7	17.9	
	65.00	8	10.3	10.3	28.2	
	67.00	4	5.1	5.1	33.3	
	68.00	1	1.3	1.3	34.6	
	70.00	7	9.0	9.0	43.6	
	71.00	1	1.3	1.3	44.9	
	72.00	10	12.8	12.8	57.7	
	74.00	8	10.3	10.3	67.9	
	76.00	10	12.8	12.8	80.8	
	78.00	6	7.7	7.7	88.5	
	80.00	3	3.8	3.8	92.3	
	83.00	4	5.1	5.1	97.4	
	85.00	2	2.6	2.6	100.0	
	Total	78	100.0	100.0		
FREQUENCIES VARIABLES=RMC_Pre RMC_Post1 RMC_Post2 RMC_Post3
  /STATISTICS=STDDEV MINIMUM MAXIMUM MEAN
  /ORDER=ANALYSIS.

Frequencies
Notes	
Output Created	31-DEC-2022 20:39:08	
Comments		
Input	Data	C:\7. Disk Terpakai INDUK\Penelitian\Penelitian 2021\Laporan Tahun Ke 2 2022\Data Hasil Pelatihan ILATAGANA.sav	
	Active Dataset	DataSet1	
	Filter	<none>	
	Weight	<none>	
	Split File	<none>	
	N of Rows in Working Data File	78	
Missing Value Handling	Definition of Missing	User-defined missing values are treated as missing.	
	Cases Used	Statistics are based on all cases with valid data.	
Syntax	FREQUENCIES VARIABLES=RMC_Pre RMC_Post1 RMC_Post2 RMC_Post3
  /STATISTICS=STDDEV MINIMUM MAXIMUM MEAN
  /ORDER=ANALYSIS.	
Resources	Processor Time	00:00:00,00	
	Elapsed Time	00:00:00,01	


Statistics	
	RMC_Pre	RMC_Post1	RMC_Post2	RMC_Post3	
N	Valid	78	78	78	78	
	Missing	0	0	0	0	
Mean	51.5256	71.3077	75.3462	75.0897	
Std. Deviation	22.14869	8.51478	6.43010	7.22223	
Minimum	4.00	46.00	64.00	46.00	
Maximum	82.00	89.00	89.00	89.00	
Frequency Table
RMC_Pre	
	Frequency	Percent	Valid Percent	Cumulative Percent	
Valid	4.00	2	2.6	2.6	2.6	
	7.00	1	1.3	1.3	3.8	
	11.00	3	3.8	3.8	7.7	
	13.00	1	1.3	1.3	9.0	
	14.00	2	2.6	2.6	11.5	
	18.00	2	2.6	2.6	14.1	
	21.00	1	1.3	1.3	15.4	
	25.00	1	1.3	1.3	16.7	
	29.00	3	3.8	3.8	20.5	
	36.00	8	10.3	10.3	30.8	
	39.00	3	3.8	3.8	34.6	
	43.00	1	1.3	1.3	35.9	
	46.00	1	1.3	1.3	37.2	
	50.00	1	1.3	1.3	38.5	
	54.00	2	2.6	2.6	41.0	
	57.00	4	5.1	5.1	46.2	
	61.00	10	12.8	12.8	59.0	
	64.00	7	9.0	9.0	67.9	
	68.00	7	9.0	9.0	76.9	
	71.00	9	11.5	11.5	88.5	
	75.00	3	3.8	3.8	92.3	
	79.00	3	3.8	3.8	96.2	
	82.00	3	3.8	3.8	100.0	
	Total	78	100.0	100.0		
RMC_Post1	
	Frequency	Percent	Valid Percent	Cumulative Percent	
Valid	46.00	1	1.3	1.3	1.3	
	54.00	1	1.3	1.3	2.6	
	61.00	11	14.1	14.1	16.7	
	64.00	10	12.8	12.8	29.5	
	68.00	10	12.8	12.8	42.3	
	71.00	14	17.9	17.9	60.3	
	75.00	9	11.5	11.5	71.8	
	78.00	1	1.3	1.3	73.1	
	79.00	8	10.3	10.3	83.3	
	82.00	8	10.3	10.3	93.6	
	86.00	3	3.8	3.8	97.4	
	89.00	2	2.6	2.6	100.0	
	Total	78	100.0	100.0		
RMC_Post2	
	Frequency	Percent	Valid Percent	Cumulative Percent	
Valid	64.00	7	9.0	9.0	9.0	
	68.00	7	9.0	9.0	17.9	
	71.00	17	21.8	21.8	39.7	
	75.00	14	17.9	17.9	57.7	
	79.00	12	15.4	15.4	73.1	
	82.00	16	20.5	20.5	93.6	
	86.00	3	3.8	3.8	97.4	
	89.00	2	2.6	2.6	100.0	
	Total	78	100.0	100.0		
RMC_Post3	
	Frequency	Percent	Valid Percent	Cumulative Percent	
Valid	46.00	1	1.3	1.3	1.3	
	64.00	5	6.4	6.4	7.7	
	68.00	9	11.5	11.5	19.2	
	71.00	18	23.1	23.1	42.3	
	75.00	12	15.4	15.4	57.7	
	79.00	13	16.7	16.7	74.4	
	82.00	13	16.7	16.7	91.0	
	86.00	5	6.4	6.4	97.4	
	89.00	2	2.6	2.6	100.0	
	Total	78	100.0	100.0		
T-TEST PAIRS=KAP_Pre WITH KAP_Post1 (PAIRED)
  /CRITERIA=CI(.9500)
  /MISSING=ANALYSIS.
T-Test
Notes	
Output Created	01-JAN-2023 03:43:59	
Comments		
Input	Data	C:\7. Disk Terpakai INDUK\Penelitian\Penelitian 2021\Laporan Tahun Ke 2 2022\Data Hasil Pelatihan ILATAGANA.sav	
	Active Dataset	DataSet1	
	Filter	<none>	
	Weight	<none>	
	Split File	<none>	
	N of Rows in Working Data File	78	
Missing Value Handling	Definition of Missing	User defined missing values are treated as missing.	
	Cases Used	Statistics for each analysis are based on the cases with no missing or out-of-range data for any variable in the analysis.	
Syntax	T-TEST PAIRS=KAP_Pre WITH KAP_Post1 (PAIRED)
  /CRITERIA=CI(.9500)
  /MISSING=ANALYSIS.	
Resources	Processor Time	00:00:00,00	
	Elapsed Time	00:00:00,01	
Paired Samples Statistics	
	Mean	N	Std. Deviation	Std. Error Mean	
Pair 1	KAP_Pre	53.8077	78	15.51878	1.75716	
	KAP_Post1	63.5641	78	11.72918	1.32807	
Paired Samples Correlations	
	N	Correlation	Sig.	
Pair 1	KAP_Pre & KAP_Post1	78	.780	.000	


Paired Samples Test	
	Paired Differences	t	df	Sig. (2-tailed)	
	Mean	Std. Deviation	Std. Error Mean	95% Confidence Interval of the Difference				
				Lower	Upper				
Pair 1	KAP_Pre - KAP_Post1	-9.75641	9.71969	1.10054	-11.94786	-7.56496	-8.865	77	.000	

T-Test
Notes	
Output Created	01-JAN-2023 05:16:12	
Comments		
Input	Data	C:\7. Disk Terpakai INDUK\Penelitian\Penelitian 2021\Laporan Tahun Ke 2 2022\Data Hasil Pelatihan ILATAGANA.sav	
	Active Dataset	DataSet1	
	Filter	<none>	
	Weight	<none>	
	Split File	<none>	
	N of Rows in Working Data File	78	
Missing Value Handling	Definition of Missing	User defined missing values are treated as missing.	
	Cases Used	Statistics for each analysis are based on the cases with no missing or out-of-range data for any variable in the analysis.	
Syntax	T-TEST GROUPS=KESIAPSIAGAAN(1 2)
  /MISSING=ANALYSIS
  /VARIABLES=KAP
  /CRITERIA=CI(.95).	
Resources	Processor Time	00:00:00,00	
	Elapsed Time	00:00:00,01	


Group Statistics	
	KESIAPSIAGAAN	N	Mean	Std. Deviation	Std. Error Mean	
KAP	1.00	0a	.	.	.	
	2.00	0a	.	.	.	
	
T-TEST GROUPS=Kesiapan_Range12(1 2)
  /MISSING=ANALYSIS
  /VARIABLES=KAP_Range12
  /CRITERIA=CI(.95).

T-Test
Notes	
Output Created	01-JAN-2023 05:18:26	
Comments		
Input	Data	C:\7. Disk Terpakai INDUK\Penelitian\Penelitian 2021\Laporan Tahun Ke 2 2022\Data Hasil Pelatihan ILATAGANA.sav	
	Active Dataset	DataSet1	
	Filter	<none>	
	Weight	<none>	
	Split File	<none>	
	N of Rows in Working Data File	78	
Missing Value Handling	Definition of Missing	User defined missing values are treated as missing.	
	Cases Used	Statistics for each analysis are based on the cases with no missing or out-of-range data for any variable in the analysis.	
Syntax	T-TEST GROUPS=Kesiapan_Range12(1 2)
  /MISSING=ANALYSIS
  /VARIABLES=KAP_Range12
  /CRITERIA=CI(.95).	
Resources	Processor Time	00:00:00,00	
	Elapsed Time	00:00:00,00	


Group Statistics	
	Kesiapan_Range12	N	Mean	Std. Deviation	Std. Error Mean	
KAP_Range12	1.00	0a	.	.	.	
	2.00	0a	.	.	.	
a. t cannot be computed because at least one of the groups is empty.	

T-TEST
  /TESTVAL=0
  /MISSING=ANALYSIS
  /VARIABLES=Kesiapan_Range12 KAP_Range12
  /CRITERIA=CI(.95).

T-Test
Notes	
Output Created	01-JAN-2023 05:23:34	
Comments		
Input	Data	C:\7. Disk Terpakai INDUK\Penelitian\Penelitian 2021\Laporan Tahun Ke 2 2022\Data Hasil Pelatihan ILATAGANA.sav	
	Active Dataset	DataSet1	
	Filter	<none>	
	Weight	<none>	
	Split File	<none>	
	N of Rows in Working Data File	78	
Missing Value Handling	Definition of Missing	User defined missing values are treated as missing.	
	Cases Used	Statistics for each analysis are based on the cases with no missing or out-of-range data for any variable in the analysis.	
Syntax	T-TEST
  /TESTVAL=0
  /MISSING=ANALYSIS
  /VARIABLES=Kesiapan_Range12 KAP_Range12
  /CRITERIA=CI(.95).	
Resources	Processor Time	00:00:00,00	
	Elapsed Time	00:00:00,01	


One-Sample Statistics	
	N	Mean	Std. Deviation	Std. Error Mean	
Kesiapan_Range12	78	15.4808	9.32571	1.05593	
KAP_Range12	78	9.7564	9.71969	1.10054	
One-Sample Test	
	Test Value = 0	
	t	df	Sig. (2-tailed)	Mean Difference	95% Confidence Interval of the Difference	
					Lower	Upper	
Kesiapan_Range12	14.661	77	.000	15.48077	13.3781	17.5834	
KAP_Range12	8.865	77	.000	9.75641	7.5650	11.9479	

T-TEST GROUPS=Kesiapan_Range12(75)
  /MISSING=ANALYSIS
  /VARIABLES=KAP_Range12
  /CRITERIA=CI(.95).

T-Test
Notes	
Output Created	01-JAN-2023 05:29:00	
Comments		
Input	Data	C:\7. Disk Terpakai INDUK\Penelitian\Penelitian 2021\Laporan Tahun Ke 2 2022\Data Hasil Pelatihan ILATAGANA.sav	
	Active Dataset	DataSet1	
	Filter	<none>	
	Weight	<none>	
	Split File	<none>	
	N of Rows in Working Data File	78	
Missing Value Handling	Definition of Missing	User defined missing values are treated as missing.	
	Cases Used	Statistics for each analysis are based on the cases with no missing or out-of-range data for any variable in the analysis.	
Syntax	T-TEST GROUPS=Kesiapan_Range12(75)
  /MISSING=ANALYSIS
  /VARIABLES=KAP_Range12
  /CRITERIA=CI(.95).	
Resources	Processor Time	00:00:00,00	
	Elapsed Time	00:00:00,01	

Group Statistics	
	Kesiapan_Range12	N	Mean	Std. Deviation	Std. Error Mean	
KAP_Range12	>= 75,00	0a	.	.	.	
	< 75,00	78	9.7564	9.71969	1.10054	
a. t cannot be computed because at least one of the groups is empty.	
CORRELATIONS
  /VARIABLES=Kesiapan_Range12 KAP_Range12
  /PRINT=TWOTAIL NOSIG
  /MISSING=PAIRWISE.
Correlations
Notes	
Output Created	01-JAN-2023 05:33:23	
Comments		
Input	Data	C:\7. Disk Terpakai INDUK\Penelitian\Penelitian 2021\Laporan Tahun Ke 2 2022\Data Hasil Pelatihan ILATAGANA.sav	
	Active Dataset	DataSet1	
	Filter	<none>	
	Weight	<none>	
	Split File	<none>	
	N of Rows in Working Data File	78	
Missing Value Handling	Definition of Missing	User-defined missing values are treated as missing.	
	Cases Used	Statistics for each pair of variables are based on all the cases with valid data for that pair.	
Syntax	CORRELATIONS
  /VARIABLES=Kesiapan_Range12 KAP_Range12
  /PRINT=TWOTAIL NOSIG
  /MISSING=PAIRWISE.	
Resources	Processor Time	00:00:00,00	
	Elapsed Time	00:00:00,00	


Correlations	
	Kesiapan_Range12	KAP_Range12	
Kesiapan_Range12	Pearson Correlation	1	.476**	
	Sig. (2-tailed)		.000	
	N	78	78	
KAP_Range12	Pearson Correlation	.476**	1	
	Sig. (2-tailed)	.000		
	N	78	78	
**. Correlation is significant at the 0.01 level (2-tailed).	

CORRELATIONS
  /VARIABLES=KAP KESIAPSIAGAAN
  /PRINT=ONETAIL NOSIG
  /STATISTICS DESCRIPTIVES
  /MISSING=PAIRWISE.
Correlations
Notes	
Output Created	01-JAN-2023 05:34:22	
Comments		
Input	Data	C:\7. Disk Terpakai INDUK\Penelitian\Penelitian 2021\Laporan Tahun Ke 2 2022\Data Hasil Pelatihan ILATAGANA.sav	
	Active Dataset	DataSet1	
	Filter	<none>	
	Weight	<none>	
	Split File	<none>	
	N of Rows in Working Data File	78	
Missing Value Handling	Definition of Missing	User-defined missing values are treated as missing.	
	Cases Used	Statistics for each pair of variables are based on all the cases with valid data for that pair.	
Syntax	CORRELATIONS
  /VARIABLES=KAP KESIAPSIAGAAN
  /PRINT=ONETAIL NOSIG
  /STATISTICS DESCRIPTIVES
  /MISSING=PAIRWISE.	
Resources	Processor Time	00:00:00,00	
	Elapsed Time	00:00:00,01	
Descriptive Statistics	
	Mean	Std. Deviation	N	
KAP	53.8077	15.51878	78	
KESIAPSIAGAAN	51.8365	10.59786	78	
Correlations	
	KAP	KESIAPSIAGAAN	
KAP	Pearson Correlation	1	.512**	
	Sig. (1-tailed)		.000	
	N	78	78	
KESIAPSIAGAAN	Pearson Correlation	.512**	1	
	Sig. (1-tailed)	.000		
	N	78	78	
**. Correlation is significant at the 0.01 level (1-tailed).	
REGRESSION
  /DESCRIPTIVES MEAN STDDEV CORR SIG N
  /MISSING LISTWISE
  /STATISTICS COEFF OUTS R ANOVA CHANGE
  /CRITERIA=PIN(.05) POUT(.10)
  /NOORIGIN
  /DEPENDENT KESIAPSIAGAAN
  /METHOD=ENTER KAP.
Regression
Notes	
Output Created	01-JAN-2023 05:35:58	
Comments		
Input	Data	C:\7. Disk Terpakai INDUK\Penelitian\Penelitian 2021\Laporan Tahun Ke 2 2022\Data Hasil Pelatihan ILATAGANA.sav	
	Active Dataset	DataSet1	
	Filter	<none>	
	Weight	<none>	
	Split File	<none>	
	N of Rows in Working Data File	78	
Missing Value Handling	Definition of Missing	User-defined missing values are treated as missing.	
	Cases Used	Statistics are based on cases with no missing values for any variable used.	
Syntax	REGRESSION
  /DESCRIPTIVES MEAN STDDEV CORR SIG N
  /MISSING LISTWISE
  /STATISTICS COEFF OUTS R ANOVA CHANGE
  /CRITERIA=PIN(.05) POUT(.10)
  /NOORIGIN
  /DEPENDENT KESIAPSIAGAAN
  /METHOD=ENTER KAP.	
Resources	Processor Time	00:00:00,00	
	Elapsed Time	00:00:00,01	
	Memory Required	3728 bytes	
	Additional Memory Required for Residual Plots	0 bytes	
Descriptive Statistics	
	Mean	Std. Deviation	N	
KESIAPSIAGAAN	51.8365	10.59786	78	
KAP	53.8077	15.51878	78	
Correlations	
	KESIAPSIAGAAN	KAP	
Pearson Correlation	KESIAPSIAGAAN	1.000	.512	
	KAP	.512	1.000	
Sig. (1-tailed)	KESIAPSIAGAAN	.	.000	
	KAP	.000	.	
N	KESIAPSIAGAAN	78	78	
	KAP	78	78	
Variables Entered/Removeda	
Model	Variables Entered	Variables Removed	Method	
1	KAPb	.	Enter	
a. Dependent Variable: KESIAPSIAGAAN	
b. All requested variables entered.	
Model Summary	
Model	R	R Square	Adjusted R Square	Std. Error of the Estimate	Change Statistics	
					R Square Change	F Change	df1	df2	Sig. F Change	
1	.512a	.262	.252	9.16316	.262	27.000	1	76	.000	
a. Predictors: (Constant), KAP	


ANOVAa	
Model	Sum of Squares	df	Mean Square	F	Sig.	
1	Regression	2267.007	1	2267.007	27.000	.000b	
	Residual	6381.221	76	83.963			
	Total	8648.228	77				
a. Dependent Variable: KESIAPSIAGAAN	
b. Predictors: (Constant), KAP	
Coefficientsa	
Model	Unstandardized Coefficients	Standardized Coefficients	t	Sig.	
	B	Std. Error	Beta			
1	(Constant)	33.023	3.766		8.768	.000	
	KAP	.350	.067	.512	5.196	.000	
a. Dependent Variable: KESIAPSIAGAAN	

REGRESSION
  /DESCRIPTIVES MEAN STDDEV CORR SIG N
  /MISSING LISTWISE
  /STATISTICS COEFF OUTS CI(95) R ANOVA CHANGE
  /CRITERIA=PIN(.05) POUT(.10)
  /NOORIGIN
  /DEPENDENT KESIAPSIAGAAN
  /METHOD=ENTER PE.

Regression
Notes	
Output Created	01-JAN-2023 05:48:40	
Comments		
Input	Data	C:\7. Disk Terpakai INDUK\Penelitian\Penelitian 2021\Laporan Tahun Ke 2 2022\Data Hasil Pelatihan ILATAGANA.sav	
	Active Dataset	DataSet1	
	Filter	<none>	
	Weight	<none>	
	Split File	<none>	
	N of Rows in Working Data File	78	
Missing Value Handling	Definition of Missing	User-defined missing values are treated as missing.	
	Cases Used	Statistics are based on cases with no missing values for any variable used.	
Syntax	REGRESSION
  /DESCRIPTIVES MEAN STDDEV CORR SIG N
  /MISSING LISTWISE
  /STATISTICS COEFF OUTS CI(95) R ANOVA CHANGE
  /CRITERIA=PIN(.05) POUT(.10)
  /NOORIGIN
  /DEPENDENT KESIAPSIAGAAN
  /METHOD=ENTER PE.	
Resources	Processor Time	00:00:00,00	
	Elapsed Time	00:00:00,01	
	Memory Required	3728 bytes	
	Additional Memory Required for Residual Plots	0 bytes	
Descriptive Statistics	
	Mean	Std. Deviation	N	
KESIAPSIAGAAN	51.8365	10.59786	78	
PE	51.7692	18.68462	78	
Correlations	
	KESIAPSIAGAAN	PE	
Pearson Correlation	KESIAPSIAGAAN	1.000	.762	
	PE	.762	1.000	
Sig. (1-tailed)	KESIAPSIAGAAN	.	.000	
	PE	.000	.	
N	KESIAPSIAGAAN	78	78	
	PE	78	78	
Variables Entered/Removeda	
Model	Variables Entered	Variables Removed	Method	
1	PEb	.	Enter	
a. Dependent Variable: KESIAPSIAGAAN	
b. All requested variables entered.	
Model Summary	
Model	R	R Square	Adjusted R Square	Std. Error of the Estimate	Change Statistics	
					R Square Change	F Change	df1	df2	Sig. F Change	
1	.762a	.581	.575	6.90805	.581	105.224	1	76	.000	
a. Predictors: (Constant), PE	


ANOVAa	
Model	Sum of Squares	df	Mean Square	F	Sig.	
1	Regression	5021.421	1	5021.421	105.224	.000b	
	Residual	3626.807	76	47.721			
	Total	8648.228	77				
a. Dependent Variable: KESIAPSIAGAAN	
b. Predictors: (Constant), PE	

Coefficientsa	
Model	Unstandardized Coefficients	Standardized Coefficients	t	Sig.	95,0% Confidence Interval for B	
	B	Std. Error	Beta			Lower Bound	Upper Bound	
1	(Constant)	29.462	2.317		12.714	.000	24.847	34.077	
	PE	.432	.042	.762	10.258	.000	.348	.516	
a. Dependent Variable: KESIAPSIAGAAN	

REGRESSION
  /DESCRIPTIVES MEAN STDDEV CORR SIG N
  /MISSING LISTWISE
  /STATISTICS COEFF OUTS CI(95) R ANOVA CHANGE
  /CRITERIA=PIN(.05) POUT(.10)
  /NOORIGIN
  /DEPENDENT KESIAPSIAGAAN
  /METHOD=ENTER PE KAP EWS MRC.

Regression
Notes	
Output Created	01-JAN-2023 06:06:45	
Comments		
Input	Data	C:\7. Disk Terpakai INDUK\Penelitian\Penelitian 2021\Laporan Tahun Ke 2 2022\Data Hasil Pelatihan ILATAGANA.sav	
	Active Dataset	DataSet1	
	Filter	<none>	
	Weight	<none>	
	Split File	<none>	
	N of Rows in Working Data File	78	
Missing Value Handling	Definition of Missing	User-defined missing values are treated as missing.	
	Cases Used	Statistics are based on cases with no missing values for any variable used.	
Syntax	REGRESSION
  /DESCRIPTIVES MEAN STDDEV CORR SIG N
  /MISSING LISTWISE
  /STATISTICS COEFF OUTS CI(95) R ANOVA CHANGE
  /CRITERIA=PIN(.05) POUT(.10)
  /NOORIGIN
  /DEPENDENT KESIAPSIAGAAN
  /METHOD=ENTER PE KAP EWS MRC.	
Resources	Processor Time	00:00:00,02	
	Elapsed Time	00:00:00,01	
	Memory Required	5280 bytes	
	Additional Memory Required for Residual Plots	0 bytes	
Descriptive Statistics	
	Mean	Std. Deviation	N	
KESIAPSIAGAAN	51.8365	10.59786	78	
PE	51.7692	18.68462	78	
KAP	53.8077	15.51878	78	
EWS	50.4103	15.72857	78	
MRC	51.5385	22.12637	78	
Correlations	
	KESIAPSIAGAAN	PE	KAP	EWS	MRC	
Pearson Correlation	KESIAPSIAGAAN	1.000	.762	.512	.552	.506	
	PE	.762	1.000	.371	.413	.050	
	KAP	.512	.371	1.000	.063	-.078	
	EWS	.552	.413	.063	1.000	-.043	
	MRC	.506	.050	-.078	-.043	1.000	
Sig. (1-tailed)	KESIAPSIAGAAN	.	.000	.000	.000	.000	
	PE	.000	.	.000	.000	.333	
	KAP	.000	.000	.	.292	.248	
	EWS	.000	.000	.292	.	.353	
	MRC	.000	.333	.248	.353	.	
N	KESIAPSIAGAAN	78	78	78	78	78	
	PE	78	78	78	78	78	
	KAP	78	78	78	78	78	
	EWS	78	78	78	78	78	
	MRC	78	78	78	78	78	

Variables Entered/Removeda	
Model	Variables Entered	Variables Removed	Method	
1	MRC, EWS, KAP, PEb	.	Enter	
a. Dependent Variable: KESIAPSIAGAAN	
b. All requested variables entered.	
Model Summary	
Model	R	R Square	Adjusted R Square	Std. Error of the Estimate	Change Statistics	
					R Square Change	F Change	df1	df2	Sig. F Change	
1	.999a	.999	.999	.39096	.999	14126.937	4	73	.000	
a. Predictors: (Constant), MRC, EWS, KAP, PE	
ANOVAa	
Model	Sum of Squares	df	Mean Square	F	Sig.	
1	Regression	8637.070	4	2159.268	14126.937	.000b	
	Residual	11.158	73	.153			
	Total	8648.228	77				
a. Dependent Variable: KESIAPSIAGAAN	
b. Predictors: (Constant), MRC, EWS, KAP, PE	
Coefficientsa	
Model	Unstandardized Coefficients	Standardized Coefficients	t	Sig.	95,0% Confidence Interval for B	
	B	Std. Error	Beta			Lower Bound	Upper Bound	
1	(Constant)	-.217	.242		-.899	.372	-.699	.264	
	PE	.255	.003	.450	89.553	.000	.249	.261	
	KAP	.248	.003	.363	79.263	.000	.242	.254	
	EWS	.247	.003	.367	78.770	.000	.241	.253	
	MRC	.253	.002	.528	124.428	.000	.249	.257	
a. Dependent Variable: KESIAPSIAGAAN	
REGRESSION
  /MISSING LISTWISE
  /STATISTICS COEFF OUTS R ANOVA
  /CRITERIA=PIN(.05) POUT(.10)
  /NOORIGIN
  /DEPENDENT KESIAPSIAGAAN
  /METHOD=ENTER PE.

Regression
Notes	
Output Created	01-JAN-2023 06:11:01	
Comments		
Input	Data	C:\7. Disk Terpakai INDUK\Penelitian\Penelitian 2021\Laporan Tahun Ke 2 2022\Data Hasil Pelatihan ILATAGANA.sav	
	Active Dataset	DataSet1	
	Filter	<none>	
	Weight	<none>	
	Split File	<none>	
	N of Rows in Working Data File	78	
Missing Value Handling	Definition of Missing	User-defined missing values are treated as missing.	
	Cases Used	Statistics are based on cases with no missing values for any variable used.	
Syntax	REGRESSION
  /MISSING LISTWISE
  /STATISTICS COEFF OUTS R ANOVA
  /CRITERIA=PIN(.05) POUT(.10)
  /NOORIGIN
  /DEPENDENT KESIAPSIAGAAN
  /METHOD=ENTER PE.	
Resources	Processor Time	00:00:00,02	
	Elapsed Time	00:00:00,01	
	Memory Required	3728 bytes	
	Additional Memory Required for Residual Plots	0 bytes	
Variables Entered/Removeda	
Model	Variables Entered	Variables Removed	Method	
1	PEb	.	Enter	
a. Dependent Variable: KESIAPSIAGAAN	
b. All requested variables entered.	
Model Summary	
Model	R	R Square	Adjusted R Square	Std. Error of the Estimate	
1	.762a	.581	.575	6.90805	
a. Predictors: (Constant), PE	


ANOVAa	
Model	Sum of Squares	df	Mean Square	F	Sig.	
1	Regression	5021.421	1	5021.421	105.224	.000b	
	Residual	3626.807	76	47.721			
	Total	8648.228	77				
a. Dependent Variable: KESIAPSIAGAAN	
b. Predictors: (Constant), PE	
Coefficientsa	
Model	Unstandardized Coefficients	Standardized Coefficients	t	Sig.	
	B	Std. Error	Beta			
1	(Constant)	29.462	2.317		12.714	.000	
	PE	.432	.042	.762	10.258	.000	
a. Dependent Variable: KESIAPSIAGAAN	
REGRESSION
  /MISSING LISTWISE
  /STATISTICS COEFF OUTS R ANOVA CHANGE
  /CRITERIA=PIN(.05) POUT(.10)
  /NOORIGIN
  /DEPENDENT KESIAPSIAGAAN
  /METHOD=ENTER PE.
Regression
Notes	
Output Created	01-JAN-2023 06:13:39	
Comments		
Input	Data	C:\7. Disk Terpakai INDUK\Penelitian\Penelitian 2021\Laporan Tahun Ke 2 2022\Data Hasil Pelatihan ILATAGANA.sav	
	Active Dataset	DataSet1	
	Filter	<none>	
	Weight	<none>	
	Split File	<none>	
	N of Rows in Working Data File	78	
Missing Value Handling	Definition of Missing	User-defined missing values are treated as missing.	
	Cases Used	Statistics are based on cases with no missing values for any variable used.	
Syntax	REGRESSION
  /MISSING LISTWISE
  /STATISTICS COEFF OUTS R ANOVA CHANGE
  /CRITERIA=PIN(.05) POUT(.10)
  /NOORIGIN
  /DEPENDENT KESIAPSIAGAAN
  /METHOD=ENTER PE.	
Resources	Processor Time	00:00:00,00	
	Elapsed Time	00:00:00,01	
	Memory Required	3728 bytes	
	Additional Memory Required for Residual Plots	0 bytes	
Variables Entered/Removeda	
Model	Variables Entered	Variables Removed	Method	
1	PEb	.	Enter	
a. Dependent Variable: KESIAPSIAGAAN	
b. All requested variables entered.	
Model Summary	
Model	R	R Square	Adjusted R Square	Std. Error of the Estimate	Change Statistics	
					R Square Change	F Change	df1	df2	Sig. F Change	
1	.762a	.581	.575	6.90805	.581	105.224	1	76	.000	
a. Predictors: (Constant), PE	
ANOVAa	
Model	Sum of Squares	df	Mean Square	F	Sig.	
1	Regression	5021.421	1	5021.421	105.224	.000b	
	Residual	3626.807	76	47.721			
	Total	8648.228	77				
a. Dependent Variable: KESIAPSIAGAAN	
b. Predictors: (Constant), PE	
Coefficientsa	
Model	Unstandardized Coefficients	Standardized Coefficients	t	Sig.	
	B	Std. Error	Beta			
1	(Constant)	29.462	2.317		12.714	.000	
	PE	.432	.042	.762	10.258	.000	
a. Dependent Variable: KESIAPSIAGAAN	


REGRESSION
  /DESCRIPTIVES MEAN STDDEV CORR SIG N
  /MISSING LISTWISE
  /STATISTICS COEFF OUTS R ANOVA CHANGE
  /CRITERIA=PIN(.05) POUT(.10)
  /NOORIGIN
  /DEPENDENT KESIAPSIAGAAN
  /METHOD=ENTER PE.

Regression
Notes	
Output Created	01-JAN-2023 06:15:38	
Comments		
Input	Data	C:\7. Disk Terpakai INDUK\Penelitian\Penelitian 2021\Laporan Tahun Ke 2 2022\Data Hasil Pelatihan ILATAGANA.sav	
	Active Dataset	DataSet1	
	Filter	<none>	
	Weight	<none>	
	Split File	<none>	
	N of Rows in Working Data File	78	
Missing Value Handling	Definition of Missing	User-defined missing values are treated as missing.	
	Cases Used	Statistics are based on cases with no missing values for any variable used.	
Syntax	REGRESSION
  /DESCRIPTIVES MEAN STDDEV CORR SIG N
  /MISSING LISTWISE
  /STATISTICS COEFF OUTS R ANOVA CHANGE
  /CRITERIA=PIN(.05) POUT(.10)
  /NOORIGIN
  /DEPENDENT KESIAPSIAGAAN
  /METHOD=ENTER PE.	
Resources	Processor Time	00:00:00,00	
	Elapsed Time	00:00:00,01	
	Memory Required	3728 bytes	
	Additional Memory Required for Residual Plots	0 bytes	


Descriptive Statistics	
	Mean	Std. Deviation	N	
KESIAPSIAGAAN	51.8365	10.59786	78	
PE	51.7692	18.68462	78	
Correlations	
	KESIAPSIAGAAN	PE	
Pearson Correlation	KESIAPSIAGAAN	1.000	.762	
	PE	.762	1.000	
Sig. (1-tailed)	KESIAPSIAGAAN	.	.000	
	PE	.000	.	
N	KESIAPSIAGAAN	78	78	
	PE	78	78	
Variables Entered/Removeda	
Model	Variables Entered	Variables Removed	Method	
1	PEb	.	Enter	
a. Dependent Variable: KESIAPSIAGAAN	
b. All requested variables entered.	
Model Summary	
Model	R	R Square	Adjusted R Square	Std. Error of the Estimate	Change Statistics	
					R Square Change	F Change	df1	df2	Sig. F Change	
1	.762a	.581	.575	6.90805	.581	105.224	1	76	.000	
a. Predictors: (Constant), PE	
ANOVAa	
Model	Sum of Squares	df	Mean Square	F	Sig.	
1	Regression	5021.421	1	5021.421	105.224	.000b	
	Residual	3626.807	76	47.721			
	Total	8648.228	77				
a. Dependent Variable: KESIAPSIAGAAN	
b. Predictors: (Constant), PE	
Coefficientsa	
Model	Unstandardized Coefficients	Standardized Coefficients	t	Sig.	
	B	Std. Error	Beta			
1	(Constant)	29.462	2.317		12.714	.000	
	PE	.432	.042	.762	10.258	.000	
a. Dependent Variable: KESIAPSIAGAAN	

REGRESSION
  /DESCRIPTIVES MEAN STDDEV CORR SIG N
  /MISSING LISTWISE
  /STATISTICS COEFF OUTS R ANOVA CHANGE
  /CRITERIA=PIN(.05) POUT(.10)
  /NOORIGIN
  /DEPENDENT KESIAPSIAGAAN
  /METHOD=ENTER EWS.
Regression
Notes	
Output Created	01-JAN-2023 06:19:14	
Comments		
Input	Data	C:\7. Disk Terpakai INDUK\Penelitian\Penelitian 2021\Laporan Tahun Ke 2 2022\Data Hasil Pelatihan ILATAGANA.sav	
	Active Dataset	DataSet1	
	Filter	<none>	
	Weight	<none>	
	Split File	<none>	
	N of Rows in Working Data File	78	
Missing Value Handling	Definition of Missing	User-defined missing values are treated as missing.	
	Cases Used	Statistics are based on cases with no missing values for any variable used.	
Syntax	REGRESSION
  /DESCRIPTIVES MEAN STDDEV CORR SIG N
  /MISSING LISTWISE
  /STATISTICS COEFF OUTS R ANOVA CHANGE
  /CRITERIA=PIN(.05) POUT(.10)
  /NOORIGIN
  /DEPENDENT KESIAPSIAGAAN
  /METHOD=ENTER EWS.	
Resources	Processor Time	00:00:00,00	
	Elapsed Time	00:00:00,01	
	Memory Required	3728 bytes	
	Additional Memory Required for Residual Plots	0 bytes	
Descriptive Statistics	
	Mean	Std. Deviation	N	
KESIAPSIAGAAN	51.8365	10.59786	78	
EWS	50.4103	15.72857	78	


Correlations	
	KESIAPSIAGAAN	EWS	
Pearson Correlation	KESIAPSIAGAAN	1.000	.552	
	EWS	.552	1.000	
Sig. (1-tailed)	KESIAPSIAGAAN	.	.000	
	EWS	.000	.	
N	KESIAPSIAGAAN	78	78	
	EWS	78	78	
Variables Entered/Removeda	
Model	Variables Entered	Variables Removed	Method	
1	EWSb	.	Enter	
a. Dependent Variable: KESIAPSIAGAAN	
b. All requested variables entered.	
Model Summary	
Model	R	R Square	Adjusted R Square	Std. Error of the Estimate	Change Statistics	
					R Square Change	F Change	df1	df2	Sig. F Change	
1	.552a	.305	.296	8.89325	.305	33.347	1	76	.000	
a. Predictors: (Constant), EWS	
ANOVAa	
Model	Sum of Squares	df	Mean Square	F	Sig.	
1	Regression	2637.399	1	2637.399	33.347	.000b	
	Residual	6010.830	76	79.090			
	Total	8648.228	77				
a. Dependent Variable: KESIAPSIAGAAN	
b. Predictors: (Constant), EWS	
Coefficientsa	
Model	Unstandardized Coefficients	Standardized Coefficients	t	Sig.	
	B	Std. Error	Beta			
1	(Constant)	33.079	3.401		9.727	.000	
	EWS	.372	.064	.552	5.775	.000	
a. Dependent Variable: KESIAPSIAGAAN	


REGRESSION
  /DESCRIPTIVES MEAN STDDEV CORR SIG N
  /MISSING LISTWISE
  /STATISTICS COEFF OUTS R ANOVA CHANGE
  /CRITERIA=PIN(.05) POUT(.10)
  /NOORIGIN
  /DEPENDENT KESIAPSIAGAAN
  /METHOD=ENTER MRC.

Regression
Notes	
Output Created	01-JAN-2023 06:21:15	
Comments		
Input	Data	C:\7. Disk Terpakai INDUK\Penelitian\Penelitian 2021\Laporan Tahun Ke 2 2022\Data Hasil Pelatihan ILATAGANA.sav	
	Active Dataset	DataSet1	
	Filter	<none>	
	Weight	<none>	
	Split File	<none>	
	N of Rows in Working Data File	78	
Missing Value Handling	Definition of Missing	User-defined missing values are treated as missing.	
	Cases Used	Statistics are based on cases with no missing values for any variable used.	
Syntax	REGRESSION
  /DESCRIPTIVES MEAN STDDEV CORR SIG N
  /MISSING LISTWISE
  /STATISTICS COEFF OUTS R ANOVA CHANGE
  /CRITERIA=PIN(.05) POUT(.10)
  /NOORIGIN
  /DEPENDENT KESIAPSIAGAAN
  /METHOD=ENTER MRC.	
Resources	Processor Time	00:00:00,02	
	Elapsed Time	00:00:00,01	
	Memory Required	3728 bytes	
	Additional Memory Required for Residual Plots	0 bytes	


Descriptive Statistics	
	Mean	Std. Deviation	N	
KESIAPSIAGAAN	51.8365	10.59786	78	
MRC	51.5385	22.12637	78	
Correlations	
	KESIAPSIAGAAN	MRC	
Pearson Correlation	KESIAPSIAGAAN	1.000	.506	
	MRC	.506	1.000	
Sig. (1-tailed)	KESIAPSIAGAAN	.	.000	
	MRC	.000	.	
N	KESIAPSIAGAAN	78	78	
	MRC	78	78	
Variables Entered/Removeda	
Model	Variables Entered	Variables Removed	Method	
1	MRCb	.	Enter	
a. Dependent Variable: KESIAPSIAGAAN	
b. All requested variables entered.	
Model Summary	
Model	R	R Square	Adjusted R Square	Std. Error of the Estimate	Change Statistics	
					R Square Change	F Change	df1	df2	Sig. F Change	
1	.506a	.256	.247	9.19876	.256	26.204	1	76	.000	
a. Predictors: (Constant), MRC	
ANOVAa	
Model	Sum of Squares	df	Mean Square	F	Sig.	
1	Regression	2217.327	1	2217.327	26.204	.000b	
	Residual	6430.902	76	84.617			
	Total	8648.228	77				
a. Dependent Variable: KESIAPSIAGAAN	
b. Predictors: (Constant), MRC	
Coefficientsa	
Model	Unstandardized Coefficients	Standardized Coefficients	t	Sig.	
	B	Std. Error	Beta			
1	(Constant)	39.337	2.655		14.818	.000	
	MRC	.243	.047	.506	5.119	.000	
a. Dependent Variable: KESIAPSIAGAAN	


FREQUENCIES VARIABLES=Jenis_Kel
  /ORDER=ANALYSIS.
Frequencies
Notes	
Output Created	10-NOV-2023 10:35:17	
Comments		
Input	Data	C:\9. Kuliah S3 UNPAD\Bahan diskusi Konsultasi Komphrehensif\RENCANA PUBLISH 2\BMC NURSING JOURNAL\BMC PUBLIC HEALTH PUIBLISH\Suplementary Material_BMC\CENTRAL DATA BASED.sav	
	Active Dataset	DataSet1	
	Filter	<none>	
	Weight	<none>	
	Split File	<none>	
	N of Rows in Working Data File	78	
Missing Value Handling	Definition of Missing	User-defined missing values are treated as missing.	
	Cases Used	Statistics are based on all cases with valid data.	
Syntax	FREQUENCIES VARIABLES=Jenis_Kel
  /ORDER=ANALYSIS.	
Resources	Processor Time	00:00:00,00	
	Elapsed Time	00:00:00,02	
[DataSet1] C:\9. Kuliah S3 UNPAD\Bahan diskusi Konsultasi Komphrehensif\RENCANA PUBLISH 2\BMC NURSING JOURNAL\BMC PUBLIC HEALTH PUIBLISH\Suplementary Material_BMC\CENTRAL DATA BASED.sav
Statistics	
Jenis_Kel  	
N	Valid	78	
	Missing	0	
Jenis_Kel	
	Frequency	Percent	Valid Percent	Cumulative Percent	
Valid	L	34	43.6	43.6	43.6	
	P	44	56.4	56.4	100.0	
	Total	78	100.0	100.0		

FREQUENCIES VARIABLES=Jenis_Kel Pengalaman
  /ORDER=ANALYSIS.

Frequencies
Notes	
Output Created	10-NOV-2023 10:40:52	
Comments		
Input	Data	C:\9. Kuliah S3 UNPAD\Bahan diskusi Konsultasi Komphrehensif\RENCANA PUBLISH 2\BMC NURSING JOURNAL\BMC PUBLIC HEALTH PUIBLISH\Suplementary Material_BMC\CENTRAL DATA BASED.sav	
	Active Dataset	DataSet1	
	Filter	<none>	
	Weight	<none>	
	Split File	<none>	
	N of Rows in Working Data File	78	
Missing Value Handling	Definition of Missing	User-defined missing values are treated as missing.	
	Cases Used	Statistics are based on all cases with valid data.	
Syntax	FREQUENCIES VARIABLES=Jenis_Kel Pengalaman
  /ORDER=ANALYSIS.	
Resources	Processor Time	00:00:00,00	
	Elapsed Time	00:00:00,00	
Statistics	
	Jenis_Kel	Pengalaman	
N	Valid	78	78	
	Missing	0	0	
Frequency Table
Jenis_Kel	
	Frequency	Percent	Valid Percent	Cumulative Percent	
Valid	L	34	43.6	43.6	43.6	
	P	44	56.4	56.4	100.0	
	Total	78	100.0	100.0		
Pengalaman	
	Frequency	Percent	Valid Percent	Cumulative Percent	
Valid	Pernah	28	35.9	35.9	35.9	
	Tidak	50	64.1	64.1	100.0	
	Total	78	100.0	100.0		
Notes	
Output Created	13-NOV-2023 14:03:25	
Comments		
Input	Data	C:\9. Kuliah S3 UNPAD\Bahan diskusi Konsultasi Komphrehensif\RENCANA PUBLISH 2\BMC NURSING JOURNAL\BMC PUBLIC HEALTH PUIBLISH\Suplementary Material_BMC\CENTRAL DATA BASED.sav	
	Active Dataset	DataSet1	
	Filter	<none>	
	Weight	<none>	
	Split File	<none>	
	N of Rows in Working Data File	78	
Missing Value Handling	Definition of Missing	User-defined missing values are treated as missing.	
	Cases Used	Statistics are based on all cases with valid data.	
Syntax	FREQUENCIES VARIABLES=Usia
  /ORDER=ANALYSIS.	
Resources	Processor Time	00:00:00,00	
	Elapsed Time	00:00:00,02	
Notes	
Output Created	13-NOV-2023 14:06:05	
Comments		
Input	Data	C:\9. Kuliah S3 UNPAD\Bahan diskusi Konsultasi Komphrehensif\RENCANA PUBLISH 2\BMC NURSING JOURNAL\BMC PUBLIC HEALTH PUIBLISH\Suplementary Material_BMC\CENTRAL DATA BASED.sav	
	Active Dataset	DataSet1	
	Filter	<none>	
	Weight	<none>	
	Split File	<none>	
	N of Rows in Working Data File	78	
Missing Value Handling	Definition of Missing	User-defined missing values are treated as missing.	
	Cases Used	Statistics are based on all cases with valid data.	
Syntax	FREQUENCIES VARIABLES=Usia
  /ORDER=ANALYSIS.	
Resources	Processor Time	00:00:00,00	
	Elapsed Time	00:00:00,00	
FREQUENCIES VARIABLES=Usia
  /ORDER=ANALYSIS.
Notes	
Output Created	13-NOV-2023 14:09:19	
Comments		
Input	Data	C:\9. Kuliah S3 UNPAD\Bahan diskusi Konsultasi Komphrehensif\RENCANA PUBLISH 2\BMC NURSING JOURNAL\BMC PUBLIC HEALTH PUIBLISH\Suplementary Material_BMC\CENTRAL DATA BASED.sav	
	Active Dataset	DataSet1	
	Filter	<none>	
	Weight	<none>	
	Split File	<none>	
	N of Rows in Working Data File	78	
Missing Value Handling	Definition of Missing	User-defined missing values are treated as missing.	
	Cases Used	Statistics are based on all cases with valid data.	
Syntax	FREQUENCIES VARIABLES=Usia
  /ORDER=ANALYSIS.	
Resources	Processor Time	00:00:00,00	
	Elapsed Time	00:00:00,00	


Frequencies
Notes	
Output Created	13-NOV-2023 14:22:42	
Comments		
Input	Data	C:\9. Kuliah S3 UNPAD\Bahan diskusi Konsultasi Komphrehensif\RENCANA PUBLISH 2\BMC NURSING JOURNAL\BMC PUBLIC HEALTH PUIBLISH\Suplementary Material_BMC\CENTRAL DATA BASED.sav	
	Active Dataset	DataSet1	
	Filter	<none>	
	Weight	<none>	
	Split File	<none>	
	N of Rows in Working Data File	78	
Missing Value Handling	Definition of Missing	User-defined missing values are treated as missing.	
	Cases Used	Statistics are based on all cases with valid data.	
Syntax	FREQUENCIES VARIABLES=Usia Usia_Kat
  /ORDER=ANALYSIS.	
Resources	Processor Time	00:00:00,00	
	Elapsed Time	00:00:00,00	
Statistics	
	Usia	1	
N	Valid	78	78	
	Missing	0	0	

Frequency Table
1	
	Frequency	Percent	Valid Percent	Cumulative Percent	
Valid	1.00	5	6.4	6.4	6.4	
	2.00	18	23.1	23.1	29.5	
	3.00	14	17.9	17.9	47.4	
	4.00	24	30.8	30.8	78.2	
	5.00	17	21.8	21.8	100.0	
	Total	78	100.0	100.0		
FREQUENCIES VARIABLES=Pendidikan
  /ORDER=ANALYSIS.
Frequencies
Notes	
Output Created	13-NOV-2023 14:42:01	
Comments		
Input	Data	C:\9. Kuliah S3 UNPAD\Bahan diskusi Konsultasi Komphrehensif\RENCANA PUBLISH 2\BMC NURSING JOURNAL\BMC PUBLIC HEALTH PUIBLISH\Suplementary Material_BMC\CENTRAL DATA BASED.sav	
	Active Dataset	DataSet1	
	Filter	<none>	
	Weight	<none>	
	Split File	<none>	
	N of Rows in Working Data File	78	
Missing Value Handling	Definition of Missing	User-defined missing values are treated as missing.	
	Cases Used	Statistics are based on all cases with valid data.	
Syntax	FREQUENCIES VARIABLES=Pendidikan
  /ORDER=ANALYSIS.	
Resources	Processor Time	00:00:00,00	
	Elapsed Time	00:00:00,00	
Statistics	
Pendidikan  	
N	Valid	78	
	Missing	0	
Pendidikan	
	Frequency	Percent	Valid Percent	Cumulative Percent	
Valid	SD	40	51.3	51.3	51.3	
	SMA	9	11.5	11.5	62.8	
	SMP	21	26.9	26.9	89.7	
	TIDAK SK	8	10.3	10.3	100.0	
	Total	78	100.0	100.0		

FREQUENCIES VARIABLES=Peran
  /ORDER=ANALYSIS.
Frequencies
Notes	
Output Created	13-NOV-2023 14:51:13	
Comments		
Input	Data	C:\9. Kuliah S3 UNPAD\Bahan diskusi Konsultasi Komphrehensif\RENCANA PUBLISH 2\BMC NURSING JOURNAL\BMC PUBLIC HEALTH PUIBLISH\Suplementary Material_BMC\CENTRAL DATA BASED.sav	
	Active Dataset	DataSet1	
	Filter	<none>	
	Weight	<none>	
	Split File	<none>	
	N of Rows in Working Data File	78	
Missing Value Handling	Definition of Missing	User-defined missing values are treated as missing.	
	Cases Used	Statistics are based on all cases with valid data.	
Syntax	FREQUENCIES VARIABLES=Peran
  /ORDER=ANALYSIS.	
Resources	Processor Time	00:00:00,00	
	Elapsed Time	00:00:00,00	
Peran  	
N	Valid	78	
	Missing	0	
Peran	
	Frequency	Percent	Valid Percent	Cumulative Percent	
Valid	APARATDS	2	2.6	2.6	2.6	
	KADERKES	5	6.4	6.4	9.0	
	KARTA	7	9.0	9.0	17.9	
	RELAWAN	60	76.9	76.9	94.9	
	TOKOH	4	5.1	5.1	100.0	
	Total	78	100.0	100.0		

FREQUENCIES VARIABLES=Pekerjaan
  /ORDER=ANALYSIS.
Frequencies
Notes	
Output Created	13-NOV-2023 15:05:26	
Comments		
Input	Data	C:\9. Kuliah S3 UNPAD\Bahan diskusi Konsultasi Komphrehensif\RENCANA PUBLISH 2\BMC NURSING JOURNAL\BMC PUBLIC HEALTH PUIBLISH\Suplementary Material_BMC\CENTRAL DATA BASED.sav	
	Active Dataset	DataSet1	
	Filter	<none>	
	Weight	<none>	
	Split File	<none>	
	N of Rows in Working Data File	78	
Missing Value Handling	Definition of Missing	User-defined missing values are treated as missing.	
	Cases Used	Statistics are based on all cases with valid data.	
Syntax	FREQUENCIES VARIABLES=Pekerjaan
  /ORDER=ANALYSIS.	
Resources	Processor Time	00:00:00,00	
	Elapsed Time	00:00:00,00	
Statistics	
Pekerjaan  	
N	Valid	78	
	Missing	0	
Pekerjaan	
	Frequency	Percent	Valid Percent	Cumulative Percent	
Valid	BURUH	46	59.0	59.0	59.0	
	PELAJAR	3	3.8	3.8	62.8	
	PNS	8	10.3	10.3	73.1	
	TDKKERJA	21	26.9	26.9	100.0	
	Total	78	100.0	100.0		
